# Potential mechanisms of acupuncture treatment for rheumatoid arthritis: a study based on network topology and machine learning

**DOI:** 10.1186/s13020-025-01209-8

**Published:** 2025-10-07

**Authors:** Feiyang Li, Zhen Liu, Yuan Xu, Yi Guo, Zhifang Xu, Gongming Yuan, Jiyu Zhao, Peiyun Li, Rui Wang, Julie Howatson, Xue Li, Yongming Guo, Yinan Gong

**Affiliations:** 1 Research Center of Experimental Acupuncture Science, Tianjin University of Traditional Chinese Medicine, Tianjin City, People’s Republic of China; 2 School of Acupuncture & Moxibustion and Tuina, Tianjin University of Traditional Chinese Medicine, Tianjin City, People’s Republic of China; 3 National Clinical Research Center for Chinese Medicine Acupuncture, Tianjin City, People’s Republic of China

**Keywords:** Acupuncture, Rheumatoid arthritis, Machine learning, Network topology, Mendelian randomization

## Abstract

**Background:**

Rheumatoid arthritis (RA) is a systemic autoimmune disease that requires multitarget therapeutic strategies. Acupuncture, an integrative therapy of traditional Chinese medicine (TCM), has shown efficacy in the clinical treatment of RA, but its molecular mechanisms remain unclear.

**Purpose:**

This study systematically elucidated the holistic regulatory effects of acupuncture on RA by integrating network topology with machine learning approaches.

**Methods:**

Data on the interactions between acupuncture-affected endogenous compounds and RA-related targets were extracted from databases, and a multidimensional interaction network was constructed to map the interactions between acupuncture and RA. screened RA-related differentially expressed genes (DEGs) from the GEOdatabase that intersected with acupuncture-responsive genes. The clusterProfiler was used for KEGG/GO enrichment analysis of these DEGs, and the immune microenvironment was analyzed via the CIBERSORTx and xCell algorithms. ConsensusClusterPlus (R package) was used for unsupervised clustering to obtain DEGs. Subsequently, key genes were identified via an ensemble machine learning model (GLM/SVM/XGB/RF), and nomograms were created. Two-sample MR and colocalization analyses were applied to validate the causal relationship between core acupuncture-affected DEGs and RA risk.

**Results:**

This study identified 10 acupuncture-regulated endogenous compounds and 49 RA-related DEGs. KEGG analysis revealed that the DEGs enriched in immune pathways included the JAK/STAT pathway, which mediates inflammatory responses, the T-cell receptor signaling pathway, which is involved in T-cell differentiation, and the TNF signaling pathway. Immunome profiling via the CIBERSORT algorithm revealed that the DEGs were enriched primarily in key immune cell subpopulations, such as M1 macrophages, activated CD4⁺ T cells, Tregs, and B lymphocytes. Machine learning identified five key genes associated with immune infiltration (STAT1, GAPDH, JAK2, PTGS2, and MDM2). MR/colocalization confirmed that acupuncture-regulated STAT1 expression was positively correlated with RA genetic susceptibility, highlighting that the STAT1-mediated JAK/STAT pathway is involved in immune remodeling.

**Conclusion:**

STAT1, GAPDH, JAK2, PTGS2, and MDM2 may be potential targets for the acupuncture treatment of RA. Acupuncture may achieve systemic immune regulation by synergistically targeting multiple pathways (JAK/STAT, TNF) and immune cells (M1 macrophages, CD4^+^ T cells). This initiative integrates the holistic philosophy of TCM with the precision of AI-driven medical science.

**Supplementary Information:**

The online version contains supplementary material available at 10.1186/s13020-025-01209-8.

## Introduction

Rheumatoid arthritis (RA), a prevalent chronic inflammatory disorder, profoundly impacts patients’ quality of life through persistent joint damage and systemic complications [1]. Characterized by progressive musculoskeletal deterioration, this disease leads to functional impairment, reduced productivity, and substantial healthcare burdens [2, 3]. Epidemiological studies indicate a global prevalence of 0.5–1.0%, with peak onset between 30 and 50 Years of age and a 3:1 female predominance [4].

Current pharmacological management employs disease-modifying antirheumatic drugs (DMARDs), biologics, and anti-inflammatory agents. While these therapies demonstrate clinical efficacy, they present limitations, including adverse effects (gastrointestinal, hepatotoxicity) [5], drug resistance [6], and increased infection risk with prolonged biologic use [7]. Notably, monoclonal antibodies targeting specific cytokines (TNF-α and IL-6) have variable response rates, with some patients developing treatment resistance [8]. This therapeutic challenge stems from the complex pathogenesis of RA, which involves dysregulated immune networks rather than isolated cytokine dysfunction [9]. These clinical observations have prompted a paradigm shift in therapeutic development—from single-target approaches to multitarget strategies addressing interconnected inflammatory pathways. This transition reflects the increasing recognition of RA as a systemic immune network disorder requiring comprehensive intervention strategies.

Acupuncture, rooted in TCM’s holistic philosophy of “regulating the body as an organic whole”, has been empirically proven to alleviate RA symptoms [10–12]. However, its multitarget mechanisms—particularly immune-microenvironment modulation—are poorly understood. Traditional reductionist approaches struggle to capture the complexity of the systemic effects of acupuncture, necessitating advanced computational strategies.

Bioinformatics methods play a vital role in the study of acupuncture transformation in academia. The amount of data generated by each stage of acupuncture discovery is increasing, and the use of these data for calculation can solve the key challenges in this process [13]. Network topology analysis has proven particularly valuable for mechanistic studies and target prediction in traditional medicine [14, 15]. Machine learning algorithms such as clustering and support vector machine model (SVM) can be used to mine useful information from a large amount of TCM data, optimize TCM research design, reduce clinical research costs, and improve research quality and efficiency. Mendelian randomization (MR) research is a method that uses genetic variation as a tool variable to explore the causal relationship between risk factors and diseases. MR can effectively overcome the bias caused by confounding factors and reverse causality and provide a new causal inference method. Recent studies have emphasized the overall and multitarget effects of acupuncture on various body systems. Recent advances in network topology and artificial intelligence (AI) have offered unprecedented opportunities to decode the “multicomponent, multitarget” nature of acupuncture. By integrating topological analysis of compound-target networks with ensemble machine learning models, we can systematically identify hub genes that serve as convergence points of the systemic regulation of acupuncture. Therefore, exploring the potential mechanism of acupuncture in the treatment of RA with a new research perspective is highly valuable and highly valuable [16].

In this study, database retrieval and classification filtering were used to identify the active components of acupuncture in the treatment of RA. On the basis of a previous study by Han et al. [17], we employed network topology and machine learning to investigate the potential mechanism of acupuncture in treating RA and analyze its effectiveness. By exploring the interaction between acupuncture and disease, the therapeutic targets of acupuncture were identified, and gene expression patterns were confirmed via bioinformatics analysis of the Gene Expression Omnibus (GEO) dataset. Through GO and KEGG functional enrichment analyses, we systematically analyzed the key biological processes and signaling pathways associated with acupuncture intervention in RA, revealing the potential molecular mechanisms involved in restoring immune homeostasis through pathways such as the JAK-STAT/TNF pathway. We also identified the characteristics of immune cell infiltration in RA, providing valuable insights for future research. Additionally, machine learning algorithms have been used to screen core therapeutic targets for RA. MR analysis and colocalization analysis established causal relationships between key targets and RA, validating the impact of the key gene STAT1 on RA risk. This study developed an AI-guided comprehensive framework that not only elucidated molecular targets for acupuncture treatment of RA but also clarified the overall logic of acupuncture therapy, offering promising prospects for current research and clinical interventions.

## Materials and methods

### Collection of potentially effective active compounds produced in the body after acupuncture treatment for RA

For this analysis, we systematically searched four databases: Web of Science, PubMed, CNKI, and Wan Fang Database (last updated on June 30, 2024), using the following terms: “acupuncture”, “body acupuncture”, “electroacupuncture”, “warm needle”, “fire needle”, “blood-letting puncture”, “rheumatoid arthritis”, “arthritis”, etc. Eligible studies: Acupuncture is defined here as the stimulation of acupuncture points on the skin with or without electrical stimulation. Studies using other forms of stimulation, such as acupressure, transcutaneous electrical nerve stimulation, and laser acupuncture, were excluded. We included randomized controlled trials in humans or animals, as well as nonrandomized comparative trials (prospective and retrospective), and removed single-group observational studies that assessed outcomes prior to and following interventions. Control interventions might take the form of placebo acupuncture, sham acupuncture, no treatment, another active treatment or medication; studies that only compared different forms of acupuncture were excluded; and the outcomes of interest were reported.

**Fig. 1:**
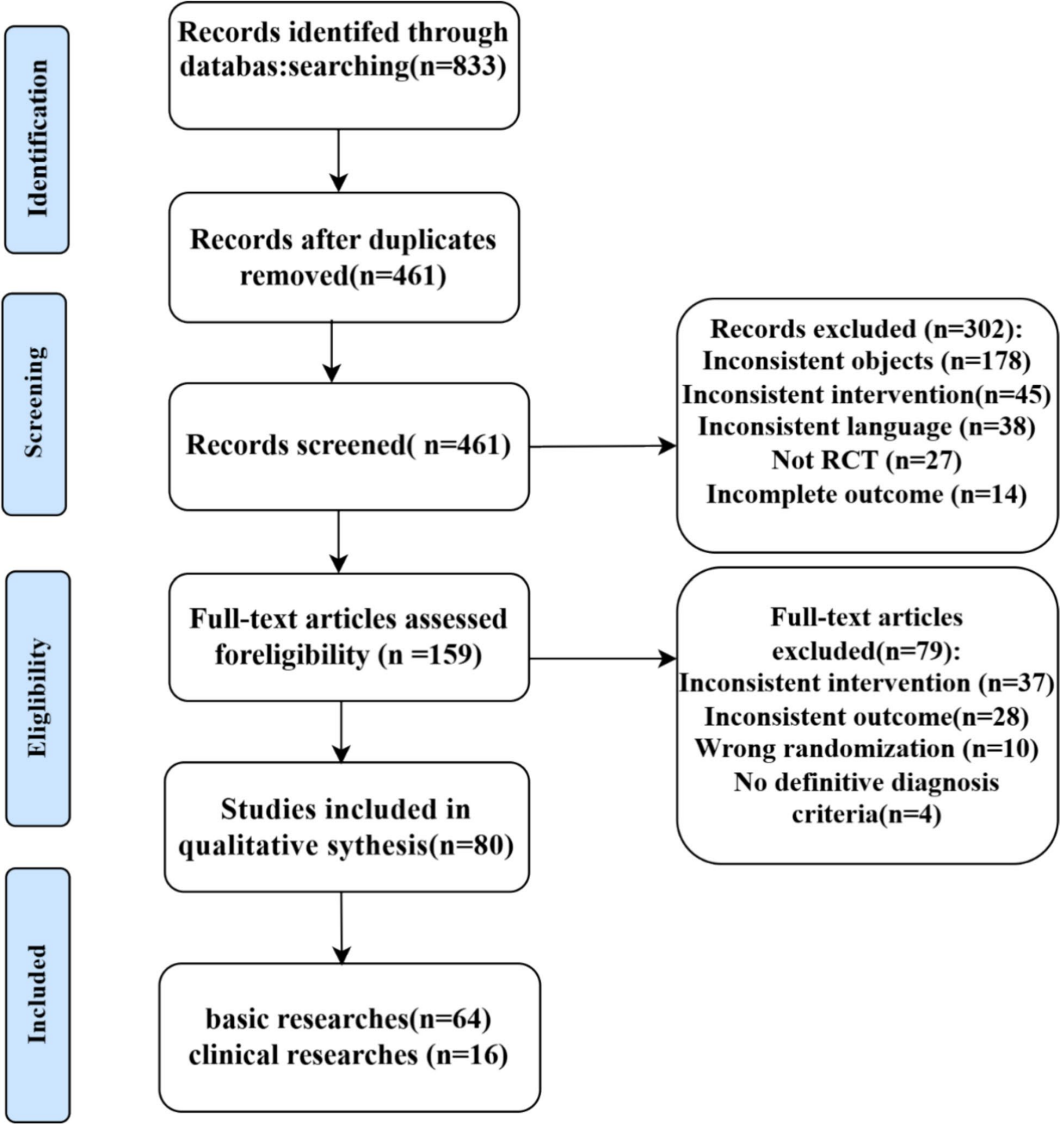
Search results and study selection

Two researchers independently searched the databases using prespecified inclusion and exclusion criteria and chose the proper literature in the full-text edition. Researchers’ controversies were addressed by discussion. If no consensus was reached, the third researcher resolved the dispute. The literature screening process is shown in Fig. 1.

### Identification of protein targets for active components produced in the body after acupuncture and acquisition of genes related to RA

The database retrieval results are systematically organized, which combines validation from multiple research types, the specificity of neuroimmune-inflammatory functions, and, on the basis of the frequency reported in the literature and the number of downstream targets, the active components generated after acupuncture are identified to ensure that they serve as core active components for acupuncture treatment of RA, with scientific validity and clinical relevance. PharmMapper is an integrated platform for pharmacophore matching that uses statistical methods to identify potential targets [18]. Swiss Target Prediction, a web server based on 2D and 3D similarity metrics and known ligand binding, accurately predicts the targets of bioactive molecules [19]. Target proteins with high binding affinity ($possibility> 0.1$) for endogenous compounds affected by acupuncture were retrieved from the PharmMapper and Swiss Target Prediction databases. The targets of the active compounds were then identified by merging the search results from the two databases and removing duplicates. Using “RA” as a keyword, relevant genes associated with RA were subsequently retrieved from two disease gene databases: GeneCards (https://www.genecards.org/) and the Comparative Toxicogenomics Database (CTD) (https://ctdbase.org/) [20, 21]. The data obtained from both databases were sorted and organized and finally entered as disease genes.

### Construction of the acupuncture-component-gene-disease network and protein‒protein interaction network

The acupuncture-related genes and RA-related genes were obtained via the online software Venn diagrams (https://bioinfogp.cnb.csic.es/tools/venny/). The intersection of these two components was used to identify effective treatment genes for acupuncture intervention in RA patients. A network was subsequently constructed via Cytoscape 3.9.0, with disease, acupuncture, components, and related genes as nodes and their relationships as edges, for topological analysis to determine the core components within the network.

STRING (Search Tool for the Retrieval of Interacting Genes/Proteins, https://cn.string-db.org/) is an online bioinformatics database aimed at providing information on gene and protein interactions [22]. The intersecting genes were imported into the STRING database with a filtering criterion of “minimum required interaction score ≥ 0.4,” and the protein‒protein interaction (PPI) network graph and Tab-Separated Values (TSV) files were downloaded and saved. Subsequently, Cytoscape software (version 3.9.0) was used for the visualization and multidimensional network construction of the PPI network for acupuncture-RA.

### Collection and preprocessing of GEO samples

The keyword “RA” was used to filter RA-related samples in the GEO database (https://www.ncbi.nlm.nih.gov/geo/). The data type was set as gene expression profiles and was limited to human samples. Gene expression matrices and clinical grouping information were collected from these samples, followed by gene symbol annotation and data correction via Perl code. This process aimed to determine the intersecting gene expression levels between acupuncture treatment for RA in the normal and RA groups in the field of network topology.

### Differences in the expression of intersection genes, chromosome location, and expression correlation of core genes

The R software packages “limma”, “heatmap”, and “ggpubr” were utilized to analyze the expression of overlapping genes in individuals with RA and in healthy individuals. Genes with a significance level of $p < 0.05$ were considered core genes, and their differential expression was visualized via box plots and heatmaps. Perl scripting was used to identify the core genes, which were then represented on a circular map. The correlation coefficient of each core gene was visually assessed via the “Rcircos” package in the R programming language.

### Differential gene enrichment analysis and expression of infiltrating immune cells in RA patient samples

To clarify the biological functions and related signaling pathways associated with the core DEGs between the RA samples and normal samples, we annotated the DEGs, aiming to understand the biological processes, molecular functions and cell compositions associated with different levels of biological functions and signaling pathways. Statistical analysis of the data, under the condition of $p < 0.05$, was carried out via R packages, including “clusterProfiler” and “enrichment”, with the results visualized in bubble diagrams. Significantly, under the criteria of $\left| {\log FC} \right|> 2$ and corrected $p < 0.05$, Venn diagrams were employed to identify DEGs by intersecting core gene clusters.

In this study, we utilized the “CIBERSORT” package in R software to conduct 1000 simulation experiments on various types of immune cells. These simulation experiments not only yield precise data on the relative composition of immune cells but also establish a benchmark for quantifying immune cell quantities. To investigate differences in immune cells further, we employed the R packages “GSVA” and “GSA Base”. These packages offer a single-sample gene set enrichment analysis (ssGSEA) method, allowing us to compare immune cell content disparities between the healthy control and RA patient groups. To delve deeper into and validate the identified core genes, we subsequently conducted a correlation test between the core genes and the ssGSEA score and visualized the correlation coefficient.

### Clustering analysis of DEGs in RA patient samples

The “ConsensusClusterPlus” package in R software was utilized to cluster RA samples on the basis of core gene expression with k-means clustering, Euclidean distance, and other algorithms, generating up to 9 clusters. The resulting clusters were assessed by comparing their expression levels through heatmaps and box plots. Additionally, principal component analysis was conducted to evaluate intercluster differences.

### Construction of a machine learning model and nomogram model for the treatment of RA with acupuncture

To gain a deeper understanding of the pivotal genes involved in acupuncture treatment for RA, we utilized the “caret” R software package to construct machine learning models, encompassing the random forest model (RF), SVM, generalized linear model (GLM), and extreme gradient boosting (XGB). RF is an ensemble machine learning technique that employs various independent decision trees to predict classification or regression [23]. The SVM algorithm is able to create a hyperplane with a maximum margin in the feature space to differentiate between positive and negative instances [24]. On the one hand, GLMs serve as an extension of multiple linear regression models, offering flexibility in evaluating the relationships between causal features of a normal distribution and categorical or continuous independent features [25]. XGB, on the other hand, consists of boosting trees on the basis of gradient boosting, enabling a careful comparison between classification error and model complexity [26]. The caret package automatically fine-tunes the parameters of these models through a grid search. All machine learning models were executed with default parameters and assessed via fivefold cross-validation. The “DALEX” package was subsequently employed to explicate the aforementioned four machine learning models, which visually represent the residual distribution and feature importance in these models. Moreover, the “pROC” R package was used to determine the area under the receiver operating characteristic (ROC) curve (AUC). Using the most effective machine learning model, we identified the top five significant variables as the key predictive genes associated with RA. Following the selection of the optimal model, we utilized the characteristic genes and their expression levels in both the normal and RA groups to create a nomogram model. Each predictor was assigned a corresponding score, with"Points” denoting the cumulative scores of the aforementioned predictors.

### MR analysis and colocalization analysis of key genes and RA

#### MR analysis

To delve deeper into the causal correlation between the key genes and susceptibility to RA, we opted for a two-sample Mendelian randomization (MR) analysis, which is particularly adept at investigating causal effects [27]. Initially, we isolated the SNPs of the characteristic genes for the exposure factors as well as the SNPs linked to RA as the outcome variables from the Integrated Epidemiological Unit (IEU) database (https://gwas.mrcieu.ac.uk/). The screening criteria for instrumental variables (SNPs) in this study are: SNPs with $p < 5e - 8$ in the exposure GWAS; exclusion of SNPs with linkage disequilibrium ($r^{2} < 0.001$) and intergenic physical distance > 10,000 kb. Based on the filtered SNPs, outcome GWAS data are extracted, and the F statistic is calculated to assess weak instrument bias (SNPs are excluded if $F < 10$). The formula for the F statistic is F = beta^2^/se^2^ (where beta is the allele effect size and se is the standard error). Additionally, SNPs with minor allele frequency $(MAF) < 0.01$ and those that have an influence on the outcome interpretation greater than the exposure are excluded to meet the assumptions of MR. Leveraging the “Two Sample MR” software package, we conducted MR analysis. Through the application of the inverse variance weighting (IVW) method, we obtained a more precise evaluation of the correlation between the expression levels of the characteristic genes and the RA risk [28]. Furthermore, Cochran’s Q statistic was employed to assess heterogeneity in the IVW outcomes, with a P value under 0.05 signifying statistically significant heterogeneity. Finally, we employed MR‒Egger regression and MR-PRESSO analysis to thoroughly evaluate potential pleiotropy [29] Any P value below 0.05 in the IVW results indicated a significant level of pleiotropy.

#### Colocalization analysis

**Fig. 2:**
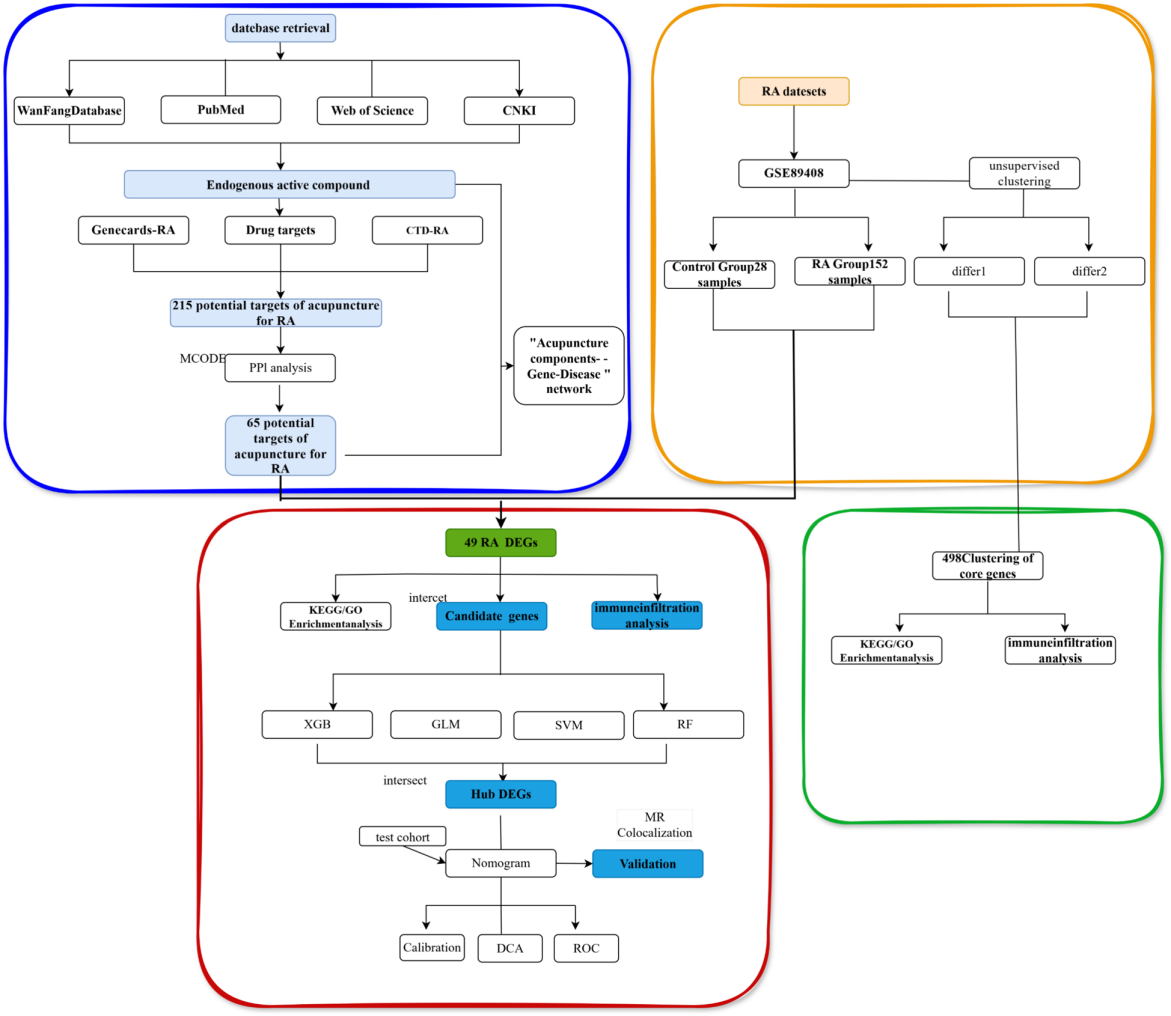
Flow chart of the study design

For genes that were significant in both cohorts, colocalization analysis of RA risk was performed via the R package coloc [30]. Analyses were performed via SNPs harmonized via the two-sample MR package with default a priori probabilities: p1 = 1E − 4, p2 = 1E − 4, and p12 = 1E − 5. P1, p2, and p12 are predefined probabilities that the SNP in the test area is substantially linked with gene expression, RA risk, or both. The posterior probabilities derived from the colocalization analysis correspond to one of five hypotheses: PPH0, SNPs are not associated with either trait; PPH1, SNPs are associated with gene expression but not with RA risk; PPH2, SNPs are associated with RA risk but not with gene expression; PPH3, SNPs associated with RA risk and gene expression but driven by different SNPs; and PPH4, SNPs associated with RA risk and gene expression, is driven by common SNPs. The threshold of significance for colocalization was set at PPH4 > 0.80, and genes that colocalize with RA could be considered potential acupuncture target genes. In the study, in order to investigate the Potential Mechanisms of the treatment of RA with Acupuncture, the core targets and mechanism of acupuncture in treating RA were predicted by Network Topology, machine learning and then validated by in Mendelian randomization and colocalization analysis (Fig. 2).

## Results

### Identification of active ingredients and related genes after acupuncture treatment and identification of RA-related genes

**Fig. 3:**
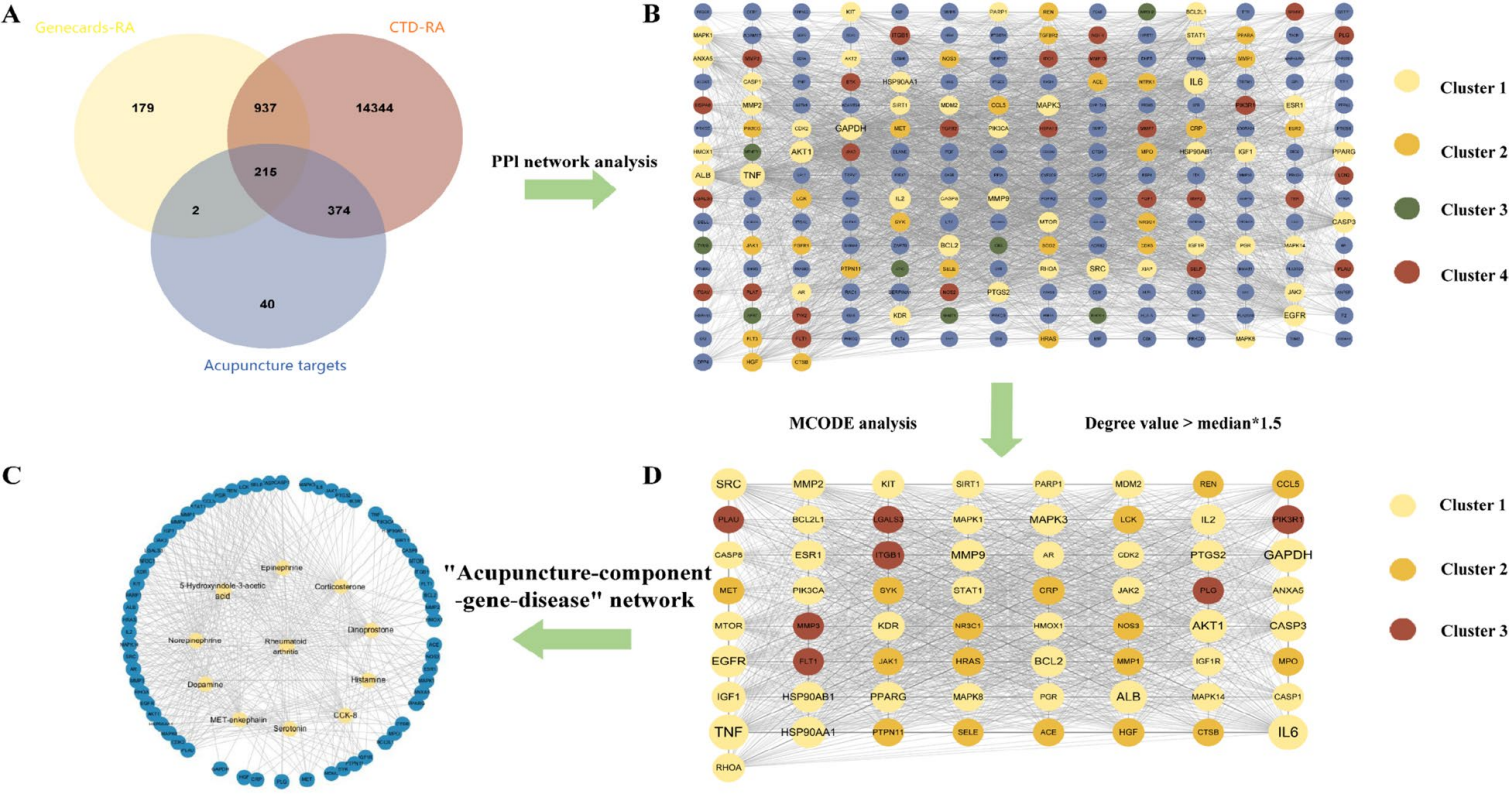
**A** Intersection diagram of Acupuncture targets and RA disease targets; **B** PPI network diagram of 215 intersection targets; **C** Further screening of the MCODE gene; **D** Acupuncture-Ingredients-Genes-Diseases diagram

By systematically searching the Web of Science, PubMed, CNKI and Wanfang databases, among the 80 included studies, the selected bioactive components were reported in at least six or more independent studies, including both animal experiments and clinical observations (Supplementary Table 1). These components are involved in neuroregulation, immunomodulation, and inflammatory responses, which aligns with the “holistic regulation” characteristics of acupuncture. Specifically, they include neurotransmitters and their metabolites, neuropeptides, hypothalamic‒pituitary axis hormones such as corticosterone (CORT), and inflammatory mediators with neuroregulatory effects, totaling 24 active components. On the basis of reports in the Literature regarding frequency, functional attributes, and the number of downstream targets, 10 compounds closely related to acupuncture treatment for RA were further selected (Supplementary Table 2). Acupuncture significantly modulated neuroimmunoregulatory molecules including neurotransmitters (serotonin, dopamine), their metabolite (5-HIAA), neuropeptides (CCK-8, MENK), hypothalamic–pituitary hormone (CORT), and the neuromodulatory inflammatory mediator dinoprostone. These compounds may ameliorate RA-associated joint inflammation and pain by attenuating hyperactive immune responses [31]. Through the PharmMapper and Swiss Target Prediction databases, a total of 631 potential target proteins associated with the active components of acupuncture were subsequently identified. Furthermore, on the basis of searches of the GeneCards and CTD databases, 1333 and 15,870 RA-related genes were obtained, respectively. Furthermore, the Venny online tool was used for intersection analysis, ultimately screening 215 common targets. These overlapping genes may constitute key molecular targets for acupuncture treatment of RA, providing crucial clues to elucidate its mechanism of action. (Fig. 3A).

### “Acupuncture-ingredient-gene-disease” network analysis and PPI network analysis

This study utilized STRING and Cytoscape software to construct a PPI network comprising 215 shared targets, consisting of 213 nodes and 3898 interaction edges, with an average connectivity of 36.601. The network was subsequently clustered into four groups via the Molecular Complex Detection (MCODE) plug-in ($k = 4$), resulting in four distinct cluster networks, as illustrated in Fig. 3B, C. Core cluster genes were identified by selecting those with a degree value ≥ 1.5 times the median in each cluster network, totaling 65 targets, such as TNF, IL6, JAK2, MAPK8, and GAPDH, and AKT1 and ALB. To delve deeper into the relationships among the central components of acupuncture, their associated genes, and RA, Cytoscape software (version 3.9.0) was used to construct an “acupuncture-component-gene-disease” network (Fig. 3D). This network comprises 76 nodes, including 10 active component nodes, 65 gene nodes, and one disease node, interconnected by 456 connections. The network topology parameters were analyzed via the Network Analyzer plug-in, which indicated an average number of adjacent nodes of 11.844, network heterogeneity of 1.085, network density of 0.156, and network centrality of 0.718. Nodes with higher degrees were identified as core nodes within the network. The most active components based on degree were dinoprostone ($Degree = 51$), CORT ($Degree = 48$), 5-HIAA ($Degree = 42$), and CCK-8 ($Degree = 40$) (Table 1). The effective components of acupuncture for RA are postulated to be those that exhibit extensive action points and robust interactions that play pivotal roles in the network. Furthermore, a single component may simultaneously affect multiple genes, reflecting multigene regulatory characteristics, and multiple components may concurrently correlate with a single gene. These findings highlight the multifaceted, multigene regulatory nature of acupuncture in treating RA.

**Table 1:** Ranking of the compounds

Name	Degree	Betweenness centrality	Closeness centrality
Dinoprostone	51	0.128465062	0.660869565
Corticosterone	48	0.093978634	0.628099174
5-Hydroxyindole-3-acetic acid	42	0.054056222	0.571428571
CCK-8	40	0.060173298	0.554744526
MET-enkephalin	37	0.037893873	0.531468531
Epinephrine	36	0.034330837	0.524137931
Norepinephrine	33	0.026687875	0.503311258
Dopamine	22	0.01489401	0.439306358
Serotonin	22	0.013884685	0.439306358
Histamine	13	0.003288627	0.397905759

Degree: The number of direct connections a node possesses within the network. A high degree indicates that the compound may interact with more potential targets and thus exhibit broader biological activity. Betweenness Centrality: Quantifies how frequently a node lies on the shortest path between any two other nodes. A high betweenness centrality suggests that the compound serves as a critical bridge linking distinct functional modules, implying a pivotal role in network regulation. Closeness Centrality: Defined as the reciprocal of the average shortest path length from the node to every other node in the network. A high closeness centrality implies that the compound can exert influence across the entire network more efficiently, potentially modulating a wider range of biological processes

### Acquisition of GEO dataset samples and correlation analysis of intersection genes

**Fig. 4:**
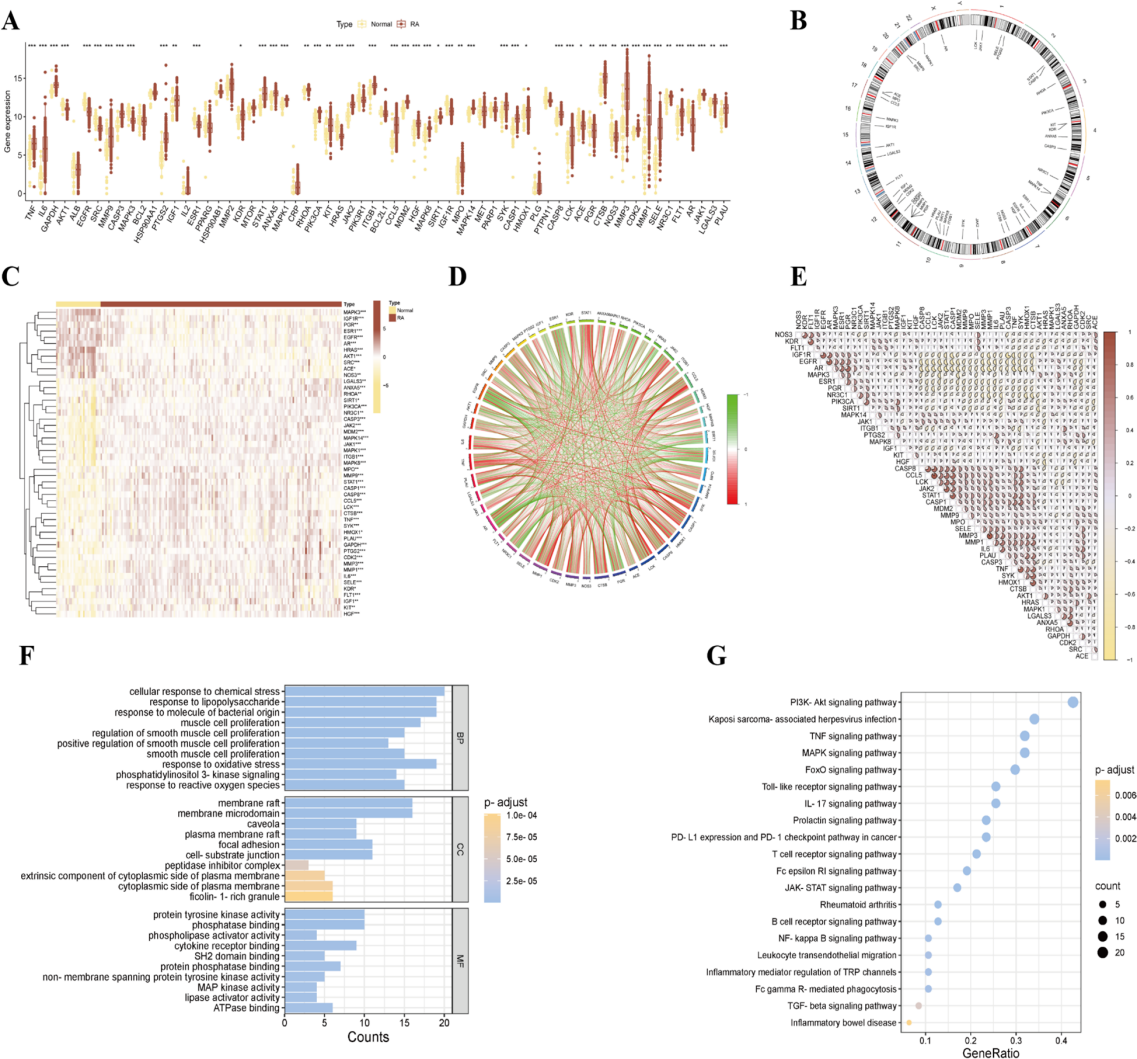
**A** Box plot of core gene expression difference analysis between normal samples and RA samples; **B** Core gene chromosome position circle diagram; **C** Heatmap of core gene expression in normal and RA samples; **D** Core gene-related network; **E** Correlation analysis between two core genes; **F** GO enrichment analysis of DEGs between normal and RA samples; **G** KEGG enrichment analysis of DEGs between normal and RA samples.($$*p < 0.05$$; $$**p < 0.01$$;$$***p < 0.001$$)

Through integrated analysis of network topology, this study identified 65 key target sites for acupuncture intervention in RA patients (Supplementary Table 3). The GSE89408 dataset related to RA was retrieved from the GEO database, which includes 28 healthy control samples and 152 RA samples. After standardization preprocessing and differential expression analysis, 49 DEGs were obtained by intersecting with the aforementioned targets, including core regulatory factors such as TNF, IL6, GAPDH, and STAT1. Clinical sample validation revealed that, in addition to the significant downregulation of nine genes (AKT1, EGFR, SRC, MAPK3, ESR1, HRAS, ACE, PGR, and NOS3) in the model group, the remaining DEGs tended to be significantly upregulated in the RA group (Fig. 4A, B). The specific chromosomal locations of acupuncture-related DEGs are shown in Fig. 4C. Correlation analysis of the DEGs in the RA samples revealed a strong relationship, as displayed in Fig. 4D, E, indicating that these genes are related to biological functions and may be involved in the same cellular processes or pathways in the disease state. This expression pattern may be used as a biomarker for disease progression or prognosis, which would be helpful in the diagnosis and treatment of RA.

### Differential gene enrichment analysis and immune cell infiltration analysis of normal samples and RA samples

Through systematic biological analysis of 49 DEGs associated with RA treated with acupuncture, this study revealed the potential mechanisms of acupuncture intervention in RA through multilevel functional annotation. Gene Ontology (GO) enrichment analysis yielded 1729 significant entries (Supplementary Table 4), with biological process (BP) terms accounting for 1582 terms, which were enriched primarily in immune stress response pathways, including lipopolysaccharide stress response, chemical stress adaptability regulation, and maintenance of redox homeostasis; molecular function (MF) terms identified 98 key entries, involving protein tyrosine kinase activity, phosphatase interaction networks, and cytokine receptor binding, among other molecular interaction features; and cellular component (CC) terms localized to 49 substructures, such as membrane raft signaling platforms, lateral plasma membrane compartments, and fibrinogen-enriched granules (Fig. 4F). Kyoto Encyclopedia of Genes and Genomes (KEGG) pathway analysis further revealed 159 significantly enriched signaling pathways, primarily involving three core regulatory modules: (1) inflammatory cascades (TNF signaling axis, NF-κB signal transduction); (2) immune cell function regulation (JAK-STAT signaling network); and (3) cellular stress adaptability pathways. (Fig. 4G) (Supplementary Table 5) These findings systematically revealed that acupuncture may exert therapeutic effects through the coordinated regulation of the inflammation‒immune‒stress triple network, providing molecular-level evidence to support its multitarget action mode.

### Unsupervised clustering of core genes in RA samples and enrichment analysis of core genes after unsupervised clustering

**Fig. 5:**
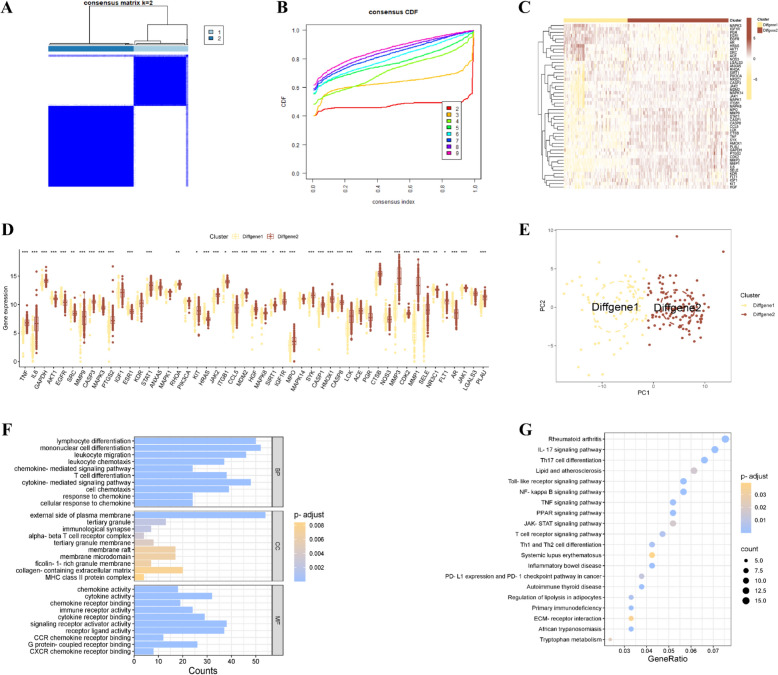
**A** Consensus cumulative distribution map of core gene sample unsupervised clustering; **B** Consensus matrix heatmap of core gene sample unsupervised clustering; **C** Heatmap of core gene expression between core gene unsupervised clusters; **D** Core gene cluster expression difference analysis box diagram; **E** PCA scatter diagram between core gene unsupervised clusters; **F** GO enrichment analysis diagram of unsupervised clustering DEGs; **G** KEGG enrichment analysis of unsupervised clustering DEGs

We performed unsupervised clustering of the samples on the basis of the core genes. This led to the identification of two primary clusters with the highest accuracy. The RA samples were stratified into Diffgene1 and Diffgene2 groups, as shown in Fig. 5A. Using cluster comparison analysis, we sought to augment and validate our previous findings (Fig. 5B). We subsequently examined the expression patterns of the core genes in the two distinct clusters, the results of which are displayed in Fig. 5C, D. Forty-nine core genes were examined in clusters Diffgene1 and Diffgene2. Notably, there were no significant differences in the expression levels of genes such as IGF1, KDR, ANXA5, MAPK1, PIK3CA, MAPK14, ACE, NOS3, or LGALS3 between the two cluster subgroups, whereas significant differences were observed in the remaining 40 genes. Principal component analysis (PCA) demonstrated the discriminative ability of the core genes between Diffgene1 and Diffgene2, as indicated in Fig. 5E.

Through functional enrichment analysis, we thoroughly investigated the biological functions and signaling pathways associated with the DEGs within the two cluster subgroups, thereby comprehensively elucidating the mechanisms of acupuncture treatment for RA. GO enrichment analysis identified 564 significantly enriched functional terms (Supplementary Table 6), of which 511 pertained to BP. These processes primarily involve immune-related processes such as lymphocyte differentiation, monocyte differentiation, and leukocyte chemotaxis. In terms of MF, 36 terms were enriched, including key molecular functions such as chemokine activity, cytokine activity, and chemokine receptor binding. CCs were enriched with 17 terms, such as the external side of the plasma membrane, tertiary granules, and immunological synapses, which are closely related to the immune response and subcellular structures (Fig. 5F). KEGG pathway analysis further revealed 38 significantly enriched signaling pathways, including the TNF signaling pathway, T-cell receptor signaling pathway, and JAK-STAT signaling pathway, among the key inflammatory regulatory pathways (Fig. 5G) (Supplementary Table 7).

Notably, both independent enrichment analyses revealed that the top 20 enriched terms were significantly associated with the immune response and inflammatory regulation processes. In the GO analysis, at the MF level, there was continuous enrichment of cytokine receptor binding and phospholipase activator activity; at the BP level, key processes such as regulation of the inflammatory response, T-cell activation, negative regulation of immune system processes, response to molecules and leukocyte migration were consistently enriched. The results of the KEGG pathway analysis also revealed high consistency, with significant enrichment of the TNF signaling pathway and the JAK-STAT signaling pathway (Figs. 4G, 5G). These findings systematically elucidate the immunomodulatory mechanisms of acupuncture in treating RA, particularly through the regulation of key inflammatory signaling pathways and the function of immune cells.

### Analysis of immune cell infiltration in normal samples and RA samples

**Fig. 6:**
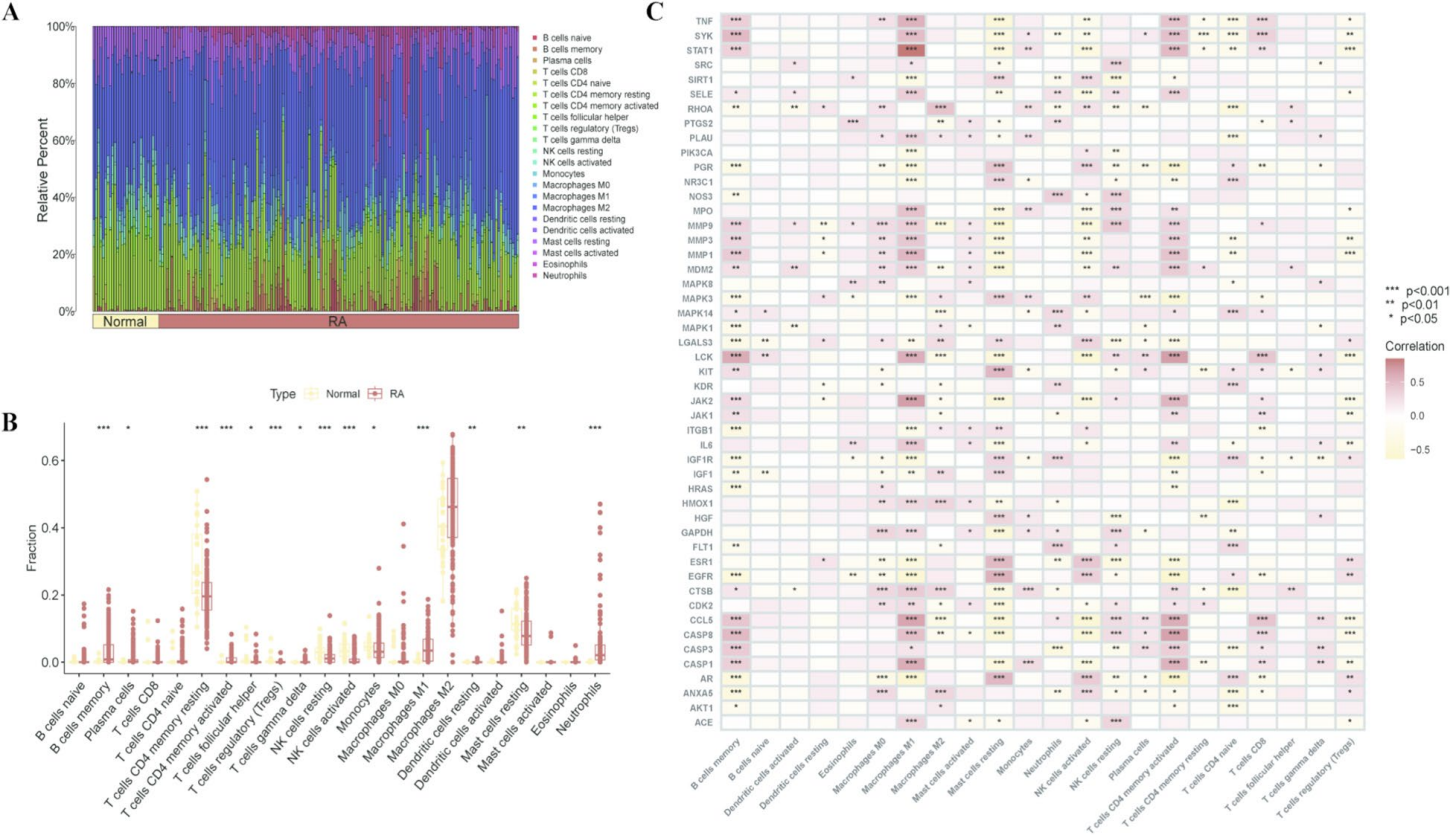
**A** The relative percentage histogram of each immune cell in the sample; **B** Box plot of the immune cell fraction of normal samples and RA samples; **C** Heatmap of the correlation analysis between core genes and immune cells ($$*p < 0.05$$; $$**p < 0.01$$; $$***p < 0.001$$)

Inflammatory cell infiltration is an important pathological feature of RA. The interaction between synovial and infiltrating cells can produce a large number of proinflammatory mediators and cytokines, which, in turn, act on the synovium and cartilage, activate nociceptors, secrete cytokines, and cause joint tissue damage. The results of the enrichment analysis of DEGs associated with the effects of acupuncture against RA showed that RA is closely related to the functions and signaling pathways of immune cells. To thoroughly investigate the differences in the immune microenvironment between RA patients and healthy individuals, this study employed the Siber sorting algorithm in R for systematic analysis of immune cell infiltration, accurately quantifying the distribution proportions of various immune cell subpopulations in the samples (Fig. 6A). Furthermore, via the ssGSEA method, significant differences in immune cell types between the two groups were identified. The results revealed 22 types of immune cells in synovial tissue, with eight subpopulations exhibiting significant differences between the RA group and the control group (Fig. 6B).

Specifically, the healthy control group presented increased abundances of resting CD4^+^ T cells, germinal center T cells, regulatory T cells (Tregs), resting and activated natural killer (NK) cells, monocytes, and resting mast cells. In contrast, the RA group presented significant increases in M1 macrophages, activated CD4^+^ T cells, memory B cells, plasma cells, resting dendritic cells, and neutrophils. Correlation analysis revealed significant associations ($p < 0.01$) between DEGs and specific immune cell subpopulations: M1 macrophages, activated CD4^+^ T cells, memory B cells, and active mast cells were positively correlated with most DEGs, such as TNF, MMP3, STAT1, IL-6, etc. In contrast, activated NK cells and resting mast cells were negatively correlated with most DEGs, such as MMP3, STAT1, JAK2, MDM2, etc. These findings further confirm the crucial regulatory role of immune cells in the pathogenesis of RA (Fig. 6C) (Supplementary Table 8).

### Machine learning model analysis and RA nomogram model construction

**Fig. 7:**
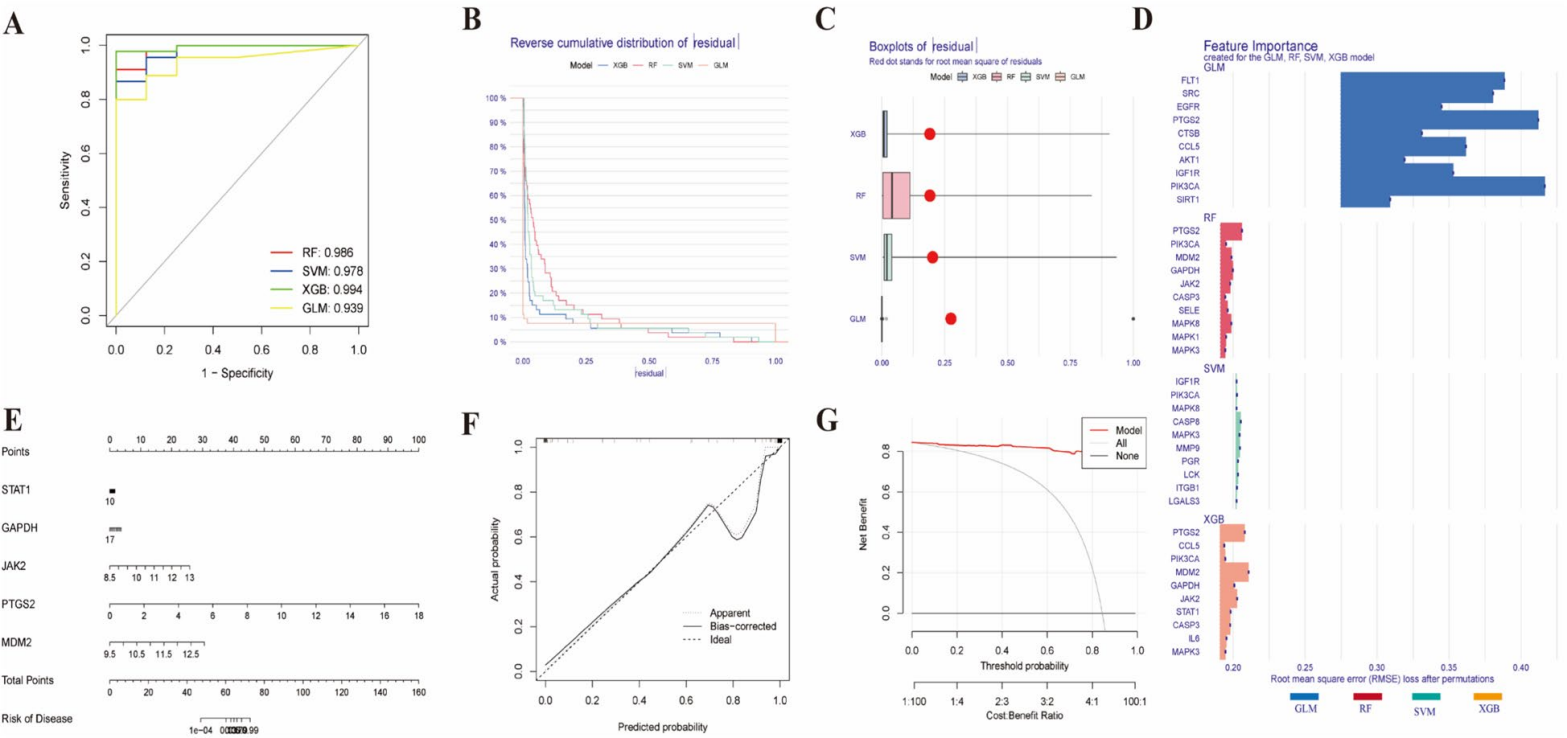
**A** ROC analysis of four machine learning models based on fivefold cross-validation in the test cohort; **B** Cumulative residual distribution of each machine learning model; boxplot; **C** Shows the residuals of each machine learning model (the red dots represent the root mean square of the residuals); **D** Important features in the RF, SVM, GLM and XGB machine models; **E** Nomogram of key genes; **F** Calibration curve of the key gene nomogram for the acupuncture treatment of RA; **G** Acupuncture treatment of the key gene nomogram decision curve of RA

This study identified 49 DEGs related to acupuncture treatment for RA as candidate genes, further identifying core DEGs influenced by acupuncture. The aim of this study was to evaluate the diagnostic potential of DEGs between RA patients and healthy individuals and to explore the impact of acupuncture on these genes. To achieve this goal, we developed four sophisticated machine learning models (RF, SVM, GLM, and XGB) to identify key genes in the RA dataset for accurate classification of patients. The DALEX software package was employed to interpret these models and visualize the residual distribution of each model in the test dataset. We evaluated the discriminatory performance of the four machine learning algorithms via fivefold cross-validation to calculate the ROC curve. Among the models, XGB demonstrated the largest AUC (GLM, AUC = 0.939; SVM, AUC = 0.978; RF, AUC = 0.986; XGB, AUC = 0.994) (Fig. 7A). The AUC value of the XGB model was verified to be 0.812 when the external dataset GSE77298 was used (Supplementary Fig. 1). Furthermore, the XGB and RF models presented relatively low residuals (Fig. 7B, C). We subsequently ranked the top ten important feature variables of each model on the basis of the root mean square error (RMSE) (Fig. 7D). In summary, the XGB model was the most adept at distinguishing distinct patient clusters. Using feature importance evaluation via the XGBoost model, this study identified five key predictive factors (STAT1, GAPDH, JAK2, PTGS2, and MDM2) as core regulatory genes.

To validate the clinical applicability of the Xgboost model, this study constructed a risk prediction nomogram based on this algorithm to quantitatively evaluate disease risk stratification in a cohort of 152 RA patients (Fig. 7E). Calibration analysis indicated good consistency between model-predicted risk and actual risk (Fig. 7F), with a Hosmer–Lemeshow test $p> 0.05$ indicating no significant calibration bias. Decision curve analysis (DCA) further confirmed that the model has significant clinical net benefit across a wide range of thresholds (Fig. 7G), with the standardized net benefit exceeding that of traditional diagnostic methods by more than 30%, indicating that this tool can serve as an effective auxiliary system for evidence-based medical decision-making, providing a reliable quantitative basis for the personalized treatment of RA.

### MR analysis and colocalization analysis results of key genes and RA

**Fig. 8:**
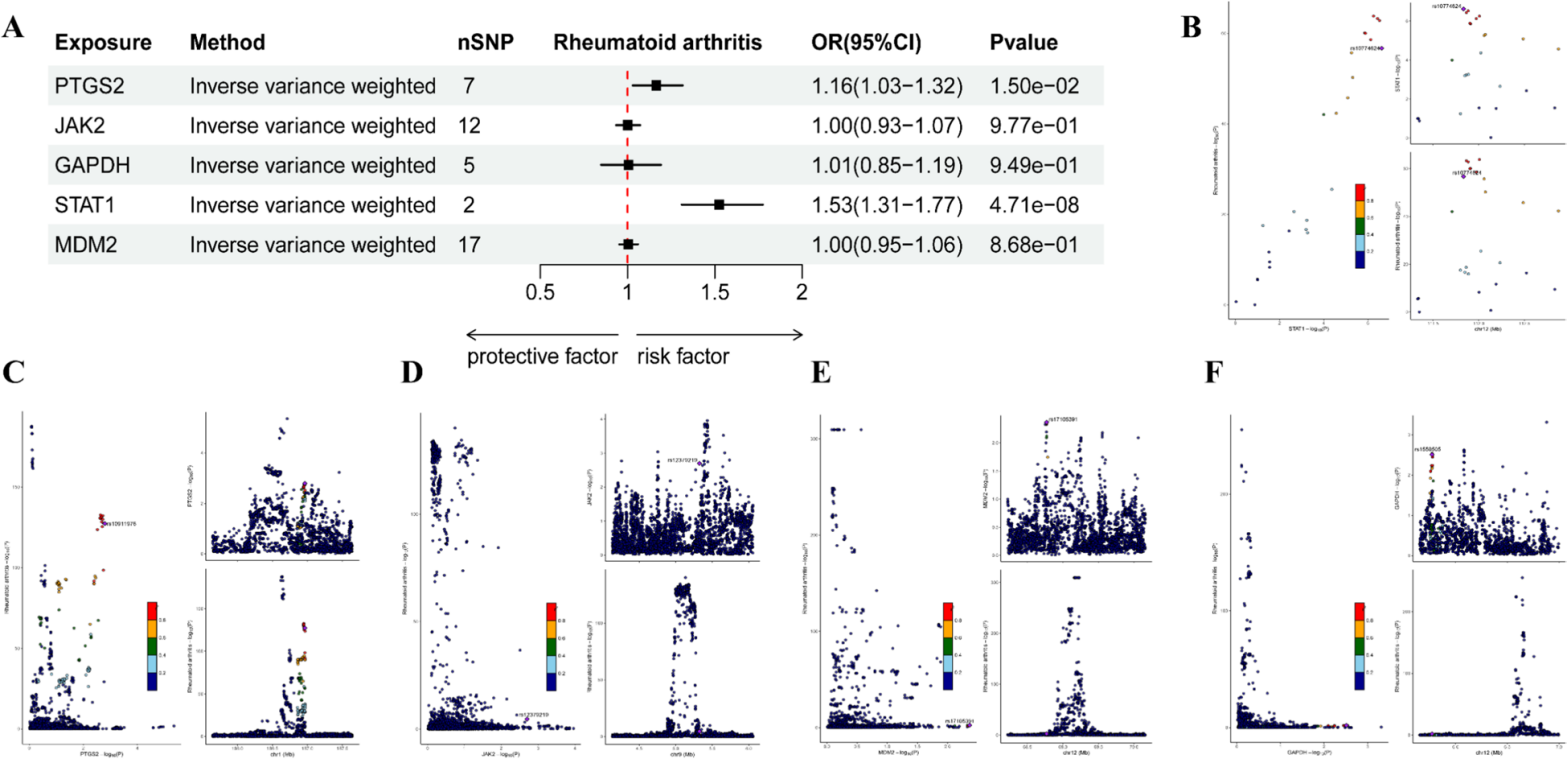
**A** Forest plot of the causal relationships between five key genes and RA via the IVW method. **B**–**F** Association of two specific single nucleotide polymorphisms (SNPs, rs10774624 and rs1077498) in the STAT1, PTGS2, JAK2, MDM2, and GAPDH genes with RA. The left diagram shows the correlation between STAT1 gene expression and RA risk. The color bar represents the log10 (*P*) value, and the darker the color is, the more significant the correlation. The right figure shows the relationship between the RA risk of these two SNP loci and their position on the chromosome

This study employs a two-sample MR framework to elucidate the causal associations between DEGs and RA. By integrating resources from the IEU Open GWAS database, which includes 14,361 RA patients and 43,923 European ancestry controls, we identified single nucleotide polymorphisms (SNPs) that meet stringent instrumental variable criteria ($p < 5e - 8$) for five key genes, including STAT1 JAK2 MDM2 PTGS2 and GAPDH (Fig. 8A). MR analysis revealed that the expression levels of STAT1 (OR = 1.53, 95% CI: 1.31–1.77, $p = 4.71e - 8$) and PTGS2 (OR = 1.16, 95% CI: 1.03–1.32, $p = 0.015$) had a significant positive causal relationship with the risk of RA (Fig. 8A). This study employed various MR methods, including IVW and MR‒Egger regression, to investigate the causal effects of the STAT1 and PTGS2 genes on RA. The robustness of these findings was confirmed through funnel plot symmetry analysis and leave-one-out analysis. Furthermore, MR‒Egger regression and MR-PRESSO analysis did not detect horizontal pleiotropy ($p> 0.05$). The Steigering test results indicated that there was no reverse causality from gene to outcome ($p < 0.05$). The heterogeneity test through IVW indicated that PTGS2 exhibited heterogeneity ($p = 0.031$). To address this, we employed the random-effects model of IVW to mitigate the impact of heterogeneity. The remaining genes showed no heterogeneity ($p> 0.05$), suggesting the reliability of the test results (Supplementary Fig. 2).

Colocalization analysis revealed a significant causal relationship between the STAT1 locus and the RA phenotype (OR = 1.53, $p = 4.71e - 8$, PPH4 > 0.8), suggesting that variations in the STAT1 gene may play a crucial role in the pathogenesis of RA (Fig. 8B). Given the crucial role of STAT1 in the immune system, interventions targeting this gene may have potential therapeutic effects on RA. Furthermore, the fact that the other four genetic loci do not share causal variants with the RA phenotype underscores the uniqueness and importance of STAT1 as a therapeutic target for RA (Fig. 8C–F). This study employs causal inference methods to systematically analyze the impact of acupuncture on gene expression in patients with RA, revealing that the expression of the STAT1 gene may be regulated. This finding not only elucidates how acupuncture might exert therapeutic effects by modulating these key genes but also offers new perspectives for developing precision treatments based on gene regulation in the future. Additionally, this study underscores the importance of integrating traditional medicine with modern scientific technology, providing valuable references for research into the treatment of other diseases.

## Discussion

In recent years, research on the molecular mechanisms of acupuncture intervention in RA has gradually become a hotspot in the field of translational medicine [32, 33]. Current evidence suggests that the active components of acupuncture can influence the pathological process of RA by modulating the dynamic balance of immune cells and the network of inflammatory factors [34]. With the advancement of high-throughput sequencing technology, integrating multiomics data with AI algorithms offers new technological pathways for elucidating the key regulatory networks of complex diseases. However, there are still limitations in the study of acupuncture mechanisms [35, 36]: first, traditional bioinformatics methods struggle to systematically reveal the patterns of multitarget synergistic effects; second, there is a lack of genetic evidence based on causal inference to support target selection.

This study systematically elucidates the molecular mechanisms of acupuncture treatment for RA by constructing a multilayer interaction network involving “acupuncture-active components-targets-disease”. Initially, 215 potential target genes were screened on the basis of network topology, with 65 target genes identified through modular analysis. Differential expression validation via the GEO dataset (GSE89408) yielded 49 DEGs that were significantly enriched in inflammation-related pathways (such as the NF-κB and JAK/STAT pathways) and immune cell regulatory networks (M1 macrophages, activated CD4^+^ T cells, Treg cells, B cells, and NK cells). A clinical prediction model based on the XGBoost algorithm (AUC = 0.994) and risk nomogram was further optimized via four machine learning algorithms to identify five core disease-related genes (STAT1, PTGS2, MDM2, GAPDH and JAK2), providing a quantitative assessment tool for the personalized treatment of RA. MR analysis confirmed that there is a significant genetic association between STAT1 and PTGS2 and the risk of RA. Colocalization analysis suggested that the STAT1 locus may be a key regulatory target for acupuncture intervention. The lack of strong evidence (PPH4 < 0.8) from the PTGS2 cohort analysis may be due primarily to the presence of multiple independent causal genetic variants within the PTGS2 locus and the relatively small effect size of PTGS2 (OR = 1.16).

This study is the first to integrate systems biology, machine learning, and causal inference methods to elucidate the network of acupuncture treatment for RA at multiple levels, including the molecular, cellular, and clinical levels. This study not only provides an innovative paradigm for modern research on traditional therapies but also lays a theoretical foundation for the development of novel targeted treatment strategies.

### Acupuncture exerts anti-inflammatory effects by regulating compounds

Dinoprostone, also referred to as prostaglandin E₂ (PGE₂), functions as a proinflammatory mediator. In RA patients, PGE₂ enhances the antigen-presenting capacity of dendritic cells (DCs) and stimulates IL-17 production by CD4⁺ αβ T cells, thereby exacerbating joint inflammation [37]. The activation of PGE₂ receptors in peripheral nociceptors contributes to pain perception. Electroacupuncture has been shown to inhibit PGE₂ receptor activity in the spinal cord of arthritis models, suppress immune cell activation within the spinal cord, block pain signal transmission, and thereby exert both anti-inflammatory and analgesic effects [38].

CORT, an endogenous glucocorticoid, has potent anti-inflammatory effects through the downregulation of proinflammatory mediators, including cyclooxygenase-2 (COX-2) and PGE_2_. Studies have demonstrated that electroacupuncture can modulate the neuroendocrine-immune response in rheumatoid arthritis rats by regulating CORT levels. Specifically, it elevates CORT concentrations to facilitate the production of anti-inflammatory cytokines such as IL-10 [39] while simultaneously activating the hypothalamic‒pituitary‒adrenal (HPA) axis to increase adrenocorticotropic hormone (ACTH) secretion [40]. This regulatory mechanism effectively suppresses neutrophil infiltration and attenuates the expression of proinflammatory cytokines, including TNF-α and IL-1β, ultimately restoring immune homeostasis.

In addition to functioning as a neurotransmitter, 5-HT is also involved in immune modulation and inflammatory responses. Through receptor-mediated signaling pathways, 5-HT can suppress the secretion of proinflammatory cytokines, including TNF-α and IL-6, thereby attenuating inflammatory reactions. Evidence suggests that acupuncture may produce analgesic effects by increasing 5-HT concentrations in the spinal cord or brain, subsequently activating the descending pain control system [41]. Following acupuncture treatment, mast cells are recruited to local acupoints via subcutaneous capillaries; upon aggregation, these mast cells undergo degranulation, releasing increased amounts of tryptase, histamine, and 5-HT, which collectively modulate immune responses and inflammatory processes [42].

Research has demonstrated that elevated concentrations of 5-HIAA, the principal metabolite of 5-HT, can ameliorate the pathological progression of RA through activation of the aryl hydrocarbon receptor (AhR) signaling pathway. This mechanism involves the suppression of germinal center B-cell differentiation into plasma cells while preserving the immunomodulatory functions of regulatory B cells (Bregs) [43]. Acupuncture has been shown to modulate the vagus nerve and central nervous system components, including the HPA axis, thereby influencing the synthesis, release, and metabolism of 5-HT and consequently regulating 5-HIAA levels [44]. Furthermore, electroacupuncture stimulation at *Jiaji* acupoints has been shown to increase both 5-HT and 5-HIAA concentrations in the spinal cord of adjuvant-induced arthritis mice, effectively mitigating inflammatory responses [45, 46].

CCK-8 is a brain-gut peptide endowed with dual neuroendocrine regulatory functions, demonstrating potent anti-inflammatory and immunomodulatory properties through its specific binding to cholecystokinin receptors [47, 48]. In the pathogenesis of RA, CCK-8 significantly inhibits the pathological activation of matrix metalloproteinases (MMPs) in synovial fibroblasts, thereby effectively mitigating joint inflammatory responses [49]. Clinical investigations have revealed that various acupuncture modalities (including cheek acupuncture and body acupuncture) can substantially upregulate CCK-8 expression levels within the central nervous system, suggesting that this may constitute a crucial mechanistic foundation for the analgesic effects of acupuncture in individuals with RA [50–52].

Enkephalins, as endogenous opioid neuropeptides, modulate immune responses and exert anti-inflammatory effects by binding to specific receptors on immune cell surfaces. Evidence suggests that acupuncture enhances enkephalin secretion via regulation of the HPA axis, a key mechanism underlying its therapeutic effects in RA [53, 54]. Experimental studies have demonstrated that acupuncture significantly elevates enkephalin levels while inhibiting pain signal transmission in arthritis models [55]. Furthermore, electroacupuncture has been shown to upregulate local enkephalin expression within joints, effectively reducing pain and inflammation in acute arthritis [56]. Collectively, these findings underscore the pivotal role of the enkephalin system in mediating the analgesic effects of acupuncture.

Catecholamine compounds (epinephrine and NE) function as both neurotransmitters and hormones. Studies have demonstrated that acupuncture exerts anti-inflammatory effects through multiple mechanisms: it stimulates adrenaline secretion from the adrenal medulla, modulates stress responses, and elevates NE levels [57]. Furthermore, electroacupuncture intervention has been shown to increase NE concentrations by activating β2-adrenergic receptors (β2-AR) in arthritis models. This mechanism effectively suppresses the expression of proinflammatory cytokines, including TNF-α, IL-1β, and IL-6, in synovial tissue, thereby significantly ameliorating synovial inflammation[58].

Dopamine exerts anti-inflammatory effects by inhibiting the synthesis of proinflammatory cytokines[59] and suppressing NLRP3 inflammasome activation [60] via D1 dopamine receptor signaling, thereby attenuating systemic inflammatory responses. Experimental studies provide compelling evidence that acupuncture modulates systemic inflammation through activation of the vagus nerve–adrenal reflex pathway, which facilitates dopaminergic neurotransmission [59].

Histamine, a biologically significant amine, is predominantly secreted by activated mast cells. In the context of immune regulation, histamine exerts modulatory effects on immune cell function by acting on four distinct receptor subtypes (H1–H4). Acupuncture therapy has been demonstrated to effectively suppress mast cell degranulation, thereby attenuating the aberrant and excessive release of histamine. Experimental studies have shown that electroacupuncture intervention markedly reduces histamine concentrations in the synovial tissue of RA model animals while concurrently inhibiting histamine synthetase activity [61]. Furthermore, acupuncture may potentiate anti-inflammatory responses mediated by H3 receptors while simultaneously mitigating proinflammatory reactions induced by excessive H1 receptor activation [42].

Preliminary research conducted by our team demonstrated that a 7-day acupuncture intervention significantly modulates PGE_2_ levels in the murine spinal cord [62]. Targeted neurotransmitter analysis further revealed that 7 days of acupuncture stimulation at the *Zusanli* (ST36) acupoint regulates multiple neural pathways, including those involving dopamine and 5-HT, consequently altering the spinal cord concentrations of 5-HT, 5-HIAA, NE, and histamine (data under review for publication). The pharmacological mechanisms of the remaining bioactive compounds will be systematically investigated through subsequent basic experimental studies and transcriptomic correlation analyses[63].

### Compounds exert anti-inflammatory effects by influencing core DEGs and immune cell function

On the basis of the multidimensional network analysis of “acupuncture-ingredient-target-diseases”, this study systematically elucidated the immunoregulatory mechanisms of acupuncture in treating RA. Network topology analysis revealed key active components, such as CCK-8, PGE_2_, and CORT, which exert their effects through synergistic action on multiple targets to regulate the immune‒inflammatory‒pain axis. Research has confirmed that acupuncture intervention can significantly regulate key neurotransmitter levels (such as by upregulating CCK-8 and enkephalin levels and downregulating PGE_2_ levels). It also involves multiple functions and signaling pathways related to the nervous system, immune response, and inflammation, which form the basis of acupuncture treatment for RA.

CCK-8 has immunomodulatory effects by suppressing Th1/Th17 cell differentiation while facilitating Treg cell induction, accompanied by diminished secretion of proinflammatory cytokines such as IL-6, which consequently attenuates JAK2 activation [64]. Furthermore, CCK-8 impedes DC maturation and B-cell-derived IgG1 production while promoting macrophage polarization toward an anti-inflammatory phenotype and stimulating the synthesis of anti-inflammatory mediators such as IL-4, collectively mitigating inflammatory responses [65, 66]. In RA studies, CCK-8 not only drives Th1 cell polarization both in vitro and in vivo but also restores Th1/Th17 homeostasis through the modulation of dendritic cell cytokine profiles (characterized by IL-12 upregulation and concurrent IL-6/IL-23 downregulation), thereby effectively ameliorating joint inflammation in animal models [67]. Notably, clinical evidence has revealed aberrant overexpression of JAK2 in peripheral blood and synovial microenvironment-resident T lymphocytes, macrophages, and fibroblast-like synoviocytes (FLSs) from RA patients [68]. CCK-8 has multiple anti-inflammatory properties, potentially promoting Th1 cell polarization and M1 macrophage differentiation by inhibiting excessive JAK2 activation, ultimately reducing the release of proinflammatory factors.

Endogenous opioids (e.g., enkephalins) modulate intricate immunoregulatory mechanisms through their interaction with μ- and δ-type opioid receptors. In a septic rat model, the binding of enkephalin to μ/δ receptors suppresses the activity and phosphorylation of JAK1/2 kinases via the Gi/o-type G protein signaling cascade, consequently disrupting downstream signal transduction and preventing the phosphorylation and nuclear translocation of STAT1 [69]. Inhibition of the STAT1 signaling pathway effectively attenuates the expression of proinflammatory mediators, including TNF-α and IL-6, while simultaneously promoting M1 macrophage polarization. Thus, enkephalin exerts its anti-inflammatory effects by inhibiting STAT1 phosphorylation, reducing proinflammatory cytokine production, and facilitating M1 macrophage polarization. In an arthritis rat model, electroacupuncture stimulation upregulated the expression of μ- and δ-type opioid receptors in the spinal cord, increased the local release of β-endorphin and enkephalin at the arthritic site and within the spinal dorsal horn, suppressed the expression of proinflammatory cytokines (TNF-α and IL-6), and alleviated hyperalgesia [70].

PTGS2, also referred to as COX-2, serves as the rate-limiting enzyme in the biosynthesis of PGE₂. Notably, PGE₂ functions both as a catalytic product of COX-2 and as a regulator of COX-2 expression and activity. Within inflammatory microenvironments, PGE₂ amplifies the production of proinflammatory mediators—including IL-6, TNF-α, and COX-2—by binding to specific receptors (EP1–EP4) and activating downstream signaling cascades such as the cAMP/PKA and NF-κB pathways, thereby establishing a positive feedback loop that sustains inflammation [71]. In rheumatoid arthritis (RA), synovial tissue often has pathologically elevated PGE₂ concentrations, which directly stimulate nociceptor terminals and exacerbate tissue damage. Furthermore, PGE₂ enhances the antigen-presenting capacity of DCs and promotes IL-17 production by CD4⁺ αβ T cells, further intensifying the inflammatory response [37, 72]. Research suggests that downregulation of the HPA axis reduces synovial PGE₂ levels in RA model rats, thereby facilitating the polarization of macrophages from a proinflammatory M1 phenotype to an anti-inflammatory M2 phenotype. This shift suppresses the expression of key proinflammatory cytokines, including IL-6, IL-1β, and TNF-α, effectively mitigating inflammation and pain [73]. In summary, the anti-inflammatory effects of acupuncture are partially mediated by its ability to reduce synovial PGE₂ levels. By diminishing PGE₂ binding to EP₂/EP₄ receptors on FLSs and macrophages, acupuncture inhibits the secretion of proinflammatory mediators and the hyperactivation of COX-2, thereby exerting its anti-inflammatory action.

Multiple immune cell subsets, including macrophages and T lymphocytes, constitutively express 5-HT receptors on their cell surfaces. Upon binding to the 5-HT_2A_ receptor, 5-HT engages a G protein-coupled receptor (GPCR)-mediated signaling cascade, wherein it directly induces tyrosine phosphorylation of JAK2, thereby activating the JAK/STAT signaling pathway [74]. Within T cells, this signaling activation upregulates the expression of 5-HT_7_ receptors, thereby increasing their responsiveness to 5-HT. As a result, 5-HT has a more pronounced inhibitory effect on T-cell proliferation and the production of proinflammatory cytokines via the 5-HT_7_ receptor [75].

In mast cells and basophils, histamine binds to H1 receptors, triggering the activation of the Gq protein-PLC-IP3/Ca^2^⁺ signaling cascade, which elevates intracellular Ca^2^⁺ levels. This Ca^2^⁺ flux subsequently induces the release of proinflammatory cytokines, including IL-4, IL-6, and TNF-α. This process further promotes the phosphorylation of JAK1/2 and the subsequent activation of STAT proteins [76]. In contrast, in macrophages and T cells, histamine engages H2 receptors, leading to the activation of the cAMP‒PKA pathway via Gs proteins. This signaling cascade suppresses STAT1 activation and attenuates the secretion of proinflammatory cytokines such as IFN-γ [77].

RA-FLSs exhibit catecholamine autocrine activity, demonstrating the capacity to synthesize and secrete dopamine. Dopamine exerts bidirectional immunomodulatory effects through binding to dopamine receptors expressed on immune cell surfaces: elevated local dopamine concentrations in the synovial microenvironment aberrantly activate T cells via D1/D5 receptor signaling, thereby amplifying autoimmune responses. Conversely, at physiological concentrations, dopamine engagement of D2 receptors suppresses macrophage phagocytic activity and attenuates proinflammatory cytokine secretion while simultaneously promoting IL-10 production [78]. Additionally, reactive oxygen species derived from dopamine metabolism can potentiate the proapoptotic function of GAPDH through S-nitrosylation. Notably, GAPDH also modulates the activity of the dopamine transporter (DAT), thereby influencing neuronal survival [79].

NE is predominantly secreted by sympathetic nerve terminals, where it exerts its physiological effects through the activation of α/β adrenergic receptors. This engagement triggers intracellular signaling cascades, including the cAMP/PKA and MAPK/ERK pathways, which markedly potentiate the proliferative, migratory, and invasive capacities of RA-FLSs. Concurrently, NE suppresses the secretion of proinflammatory cytokines such as TNF-α, IFN-γ, and IL-12, thereby mitigating localized inflammatory responses [80, 81]. Notably, emerging evidence has demonstrated that NE can effectively attenuate LPS-induced microglial inflammation by inhibiting NF-κB nuclear translocation and STAT1 phosphorylation, underscoring the pleiotropic nature of its biological effects [82].

CORT, a prototypical glucocorticoid, forms a complex with the glucocorticoid receptor (GR) in the cytoplasm, subsequently translocating to the nucleus. Through this GR-mediated mechanism, CORT modulates the expression of the circadian gene PER2 in peripheral tissues. Studies have demonstrated that PER2 sustains the immunosuppressive function of regulatory T cells (Tregs) by inhibiting MDM2. This regulatory axis becomes dysregulated in the synovial tissue of rheumatoid arthritis (RA) patients, resulting in aberrant proliferation of proinflammatory Th17 cells [83]

The functional enrichment analysis of the study components and DEGs revealed that their significant associations were predominantly enriched in key biological pathways, including “regulation of inflammatory response” and “immune cell activation”. These findings are strongly concordant with the established anti-inflammatory and immunomodulatory mechanisms of acupuncture, thereby offering substantial support for the biological plausibility underlying the component–DEG associations. Collectively, these results reveal that acupuncture exerts dynamic regulatory effects on the neuroendocrine-immune system through a multidimensional “component-target-pathway” network. Specifically, it modulates the functional state of immune cells via mediators such as CCK-8 and PGE_2_, ultimately eliciting multifaceted therapeutic effects encompassing anti-inflammatory, immunomodulatory, and analgesic actions. This comprehensive understanding establishes a robust theoretical foundation for developing mechanism-based, targeted therapeutic strategies for RA through acupuncture intervention.

### Potential mechanism of acupuncture in the treatment of RA

#### Immune cells in RA

A hallmark characteristic of the RA synovial microenvironment is the pathological accumulation of diverse immune cell populations, including T lymphocytes, B lymphocytes, macrophages, NK cells, and mast cells [84, 85]. Notably, synovial macrophages demonstrate a predominant M1 polarization phenotype during RA pathogenesis. These cells not only retain osteoclastogenic potential but also orchestrate the chemotactic recruitment of monocytes and neutrophils through the elaboration of proinflammatory cytokines. Furthermore, they play a pivotal role in T-cell activation and contribute to the dysregulated proliferation of synoviocytes, collectively sustaining a chronic inflammatory cascade [86].

Among the immune cells infiltrating the synovium, subsets of CD4^+^ T cells play a central role [87]. Activated CD4^+^ and CD8^+^ T cells and Th17 cells significantly promote osteoclast differentiation by releasing key effector molecules, such as NF-κB ligands, TNF-α, IL-1, IL-6, and IL-17. In contrast, IFN-γ and IL-4 secreted by Th1 and Th2 cells exert negative regulatory effects on osteoclastogenesis [88]. Notably, activated CD4^+^ T cells can further amplify inflammatory damage in joint tissues through cascaded activation of macrophages and B cells, whereas resting CD4^+^ T cells do not participate in this pathological process [89]. Follicular helper T (Tfh) cells within CD4^+^ T-cell subsets play a unique role in RA, as their abnormal expression of the surface markers CXCR5, ICOS, and PD1 is closely related to disease occurrence. These cells participate in autoimmune responses by regulating B-cell antibody production. Concurrently, functional defects in Tregs may disrupt immune homeostasis, leading to excessive activation of autoreactive T cells [90]. The mechanism of B-cell involvement in RA encompasses multipathway regulation: B cells promote osteoclast maturation by producing receptor activators of the NF-κB ligand [91], activate memory B cells to increase the immune response, and induce synovial tissue to produce proinflammatory cytokines such as IL-1α, IL-23, IL-12, IL-6, and TNF-α, thereby exacerbating bone destruction [92]. Mast cells, as resident components of the synovium in innate immunity, regulate T/B-cell and APC function by secreting mediators such as TNF-α, IL-1β, IL-4, and IL-5 [93]. NK cells participate in disease progression through cytotoxic activity and cytokine networks [94].

Previous studies have demonstrated that acupuncture exerts anti-inflammatory effects on joint inflammation by restoring the Th1/Treg cell balance, as indicated by reduced levels of proinflammatory cytokines (IFN-γ and IL-17) and elevated levels of anti-inflammatory cytokines (TGF-β) [10]. Acupuncture intervention significantly suppresses M1 macrophage polarization in synovial tissue while facilitating the differentiation of T cells into anti-inflammatory subsets, thereby exerting anti-inflammatory and analgesic effects through the downregulation of proinflammatory cytokines, including TNF-α, IL-6, and IL-17 [95]. Furthermore, acupuncture may ameliorate immune dysregulation in RA by modulating NK cell activity, whose pathological activation is closely linked to exacerbated joint inflammation [96]. This study employed the Siebel algorithm in R to analyze the immune cell infiltration characteristics of RA patients and revealed significant upregulation of M1 macrophages, activated CD4^+^ T cells, and memory B cells. This finding corroborates the theory of immune-mediated synovial damage, suggesting that abnormal immune responses may directly drive inflammation through metabolic remodeling and polarization changes in macrophages, while persistent activation of T cells leads to a vicious cycle of autoimmunity. In summary, different subpopulations of immune cells collectively constitute the core mechanism of RA pathogenesis through complex interaction networks.

#### Acupuncture regulates core DEGs to influence immune cell function and inhibit inflammatory responses in RA

The anti-inflammatory effects of acupuncture involve the coordinated regulation of multiple pathways, including the NF‒κB and JAK‒STAT signaling axes. In contrast, the production of TNF-α and IL-6, which are primary downstream acute-phase inflammatory factors, is often limited to a single pathway. Although they are commonly elevated in various inflammatory diseases, their specificity is relatively low. As a core effector molecule of the JAK/STAT signaling pathway, the functional regulation of STAT1 strictly depends on dual phosphorylation at Y701 and S727, which is mediated by JAK. Activated STAT1 can directly interact with the NF-κB p65 subunit, and this interaction is particularly significant in FLSs [97]. Clinical data confirm that the expression levels of STAT1 in the synovial tissue of RA patients are significantly greater than those in the OA control group and that, after effective treatment, its expression can be specifically downregulated. [98]. During the RA pathological process, cytokines such as IFN-γ and IL-6 activate the JAK1/JAK2 kinase cascade by binding to surface receptors on macrophages (e.g., IFN-γR and IL-6R), leading to the phosphorylation and activation of STAT1. Activated STAT1 upregulates HIF-1α expression, enhancing glycolytic metabolism to meet the energy demands of proinflammatory M1 macrophages, thereby driving self-sustenance and the amplification of inflammatory responses [99, 100]. Blocking STAT1 signaling effectively inhibits the expression of the proinflammatory genes TNF-α and IL-6 and promotes the degree of M1 polarization [101]. Research indicates that STAT1 signaling significantly enhances the efficiency of T-cell antigen presentation by increasing the expression of the costimulatory molecules CD80 and CD86 on B-cell surfaces, mediating abnormal intercellular interactions, and continuously exacerbating local joint inflammation [102].

JAK2 kinase, a core regulatory molecule of cytokine signaling pathways, drives disease progression through various mechanisms. Clinical studies have shown that T lymphocytes, macrophages, and FLSs in the peripheral blood and synovial microenvironment of RA patients exhibit abnormally high expression of JAK2 [68]. In T cells, proinflammatory cytokines such as IL-6 and IL-12 bind to their receptors, triggering a STAT3/STAT1 phosphorylation cascade, which promotes the specific secretion of IL-17A by Th17 cells, disrupting Treg-mediated immune balance and ultimately exacerbating synovial inflammation and cartilage destruction [103, 104]. Inhibiting the JAK/STAT pathway can promote the polarization of M1 macrophages to M2 macrophages[105]. Inhibiting JAK2 expression can also reduce the production of the proinflammatory cytokines TNF-α and IL-6, thereby alleviating local inflammatory responses[106].

Electroacupuncture stimulation of the ST36 acupoint can alleviate inflammatory injury by reducing IL-6 release and inhibiting the activation of the JAK/STAT pathway, thereby decreasing the expression of downstream signaling molecules such as TNF-α, Bax, and Caspase-3 in lung tissue and plasma[107]. Correlation analysis of the core DEGs and immune cells identified in this study revealed that JAK2/STAT1 is significantly positively correlated with M1 macrophages and CD4^+^ T cells, whereas JAK2 is significantly negatively correlated with M2 macrophages. These findings suggest that acupuncture may function by inhibiting the phosphorylation of STAT1 in the JAK2/STAT1 signaling pathway, promoting the polarization of M1 macrophages to M2 macrophages, and suppressing the excessive activation of CD4⁺ T cells, thereby correcting the imbalance of CD4⁺ T-cell subsets. Consequently, it reduces Th17-mediated immune damage and alleviates local synovial inflammation.

PTGS2 is a key enzyme in the PGE₂ biosynthetic pathway. Its activation leads to increased PGE₂ production, which is involved in the regulation of inflammation and immune responses. In states of acute inflammation, the expression of PTGS2 within macrophages increases, driving the production of prostaglandin-like substances, which promote the release of proinflammatory factors by M1-type macrophages (such as TNF-α and IL-1β). Targeted inhibition of PTGS2 activity can effectively block the generation of such inflammatory mediators [108]. The downregulation of PTGS2 expression can significantly inhibit the degranulation process of mast cells, reduce increased vascular permeability, and downregulate the expression levels of various proinflammatory factors [109]. Furthermore, inhibiting PTGS2 in FLS reduces PGE_2_ synthesis, which helps in the activation of Tregs, maintains immune tolerance status, and promotes the formation of an anti-inflammatory microenvironment [110]. This study revealed that PTGS2 has a significant positive correlation with activated mast cells and a significant negative correlation with M2 macrophages, suggesting that acupuncture may inhibit inflammation by suppressing PTGS2 activity, reducing the production of the proinflammatory prostaglandin PGE_2_, thereby inhibiting mast cell degranulation or promoting macrophage polarization toward M2-type macrophages, alleviating inflammatory responses, and providing molecular evidence for the anti-inflammatory mechanism of acupuncture in the treatment of RA.

GAPDH, a key enzyme in glycolysis, regulates cellular energy metabolism (ATP production) by catalyzing the conversion of 3-phosphoglycerdehyde to 1,3-bisphosphoglycerate. Under normal conditions, macrophages efficiently produce ATP through oxidative phosphorylation. In an inflammatory state, immune cells such as M1 macrophages and eosinophils undergo “metabolic reprogramming”, shifting from efficient energy production through oxidative phosphorylation to glycolysis. This transformation is accelerated by the activation of GAPDH, leading to rapid ATP generation and biosynthetic precursor production, which promotes the extensive synthesis of the proinflammatory cytokines TNF-α and IL-6. Additionally, the NADH generated by GAPDH-catalyzed reactions can produce reactive oxygen species (ROS) through the electron transport chain, thereby activating proinflammatory pathways such as the NF-κB and MAPK pathways [111]. Moreover, phosphoenolpyruvate, an intermediate product of glycolysis, can directly inhibit anti-inflammatory pathways such as the AMPK pathway, further amplifying the inflammatory response [112]. In addition to driving glycolysis, GAPDH directly participates in the regulation of inflammatory signaling through protein interactions. GAPDH can promote inflammasome assembly by interacting with ASC, a core component of the NLRP3 inflammasome, thereby activating caspase-1 and leading to the maturation and release of proinflammatory factors such as IL-1β [113]. This study revealed that the expression of GAPDH is elevated in RA patients and significantly positively correlated with M1 macrophages, suggesting that acupuncture may reduce the activity of GAPDH in RA, decrease the accumulation of glycolytic products, reduce the synthesis of proinflammatory cytokines such as TNF-α and IL-1β, and promote the transformation of M1 macrophages toward an anti-inflammatory M2 phenotype, thereby restoring immune homeostasis.

MDM2 is an E3 ubiquitin ligase that regulates the degradation of target proteins through ubiquitination, participating in various cellular processes, such as the cell cycle, apoptosis regulation, and glycolysis. Research has shown that, compared with those in healthy control synovial tissue, the mRNA and protein expression levels of MDM2 are significantly elevated in the synovial tissue of RA patients [114]. In an inflammatory state, MDM2 directly acts as a transcription coactivator to activate the NF-κB signaling pathway, driving the production of various proinflammatory cytokines, such as TNF-α, IL-1β, IL-6, chemokines, and MMPs, thereby exacerbating inflammation and joint destruction[115]. MDM2 extends the survival of Th17 cells by inhibiting p53-dependent apoptosis while impairing the immunosuppressive function of Tregs through the ubiquitination and degradation of Foxp3[116]. MDM2 can exacerbate inflammatory responses by integrating the iNOS-NO and HIF-1α signaling networks, and its overactivation promotes glycolysis in M1-type macrophages [117]. Specific inhibition of MDM2 levels can suppress the survival and death of inflammatory cells such as macrophages and T cells, thereby affecting their activity and alleviating inflammation [115]. This study revealed a significant positive correlation between MDM2 and M1 macrophages, suggesting that the anti-inflammatory effects of acupuncture may partially result from the downregulation of MDM2 gene expression, the inhibition of the NF-κB signaling pathway to reduce TNF-α/IL-6 production, and the suppression of glycolysis in M1 macrophages, thereby alleviating inflammation and tissue damage.

**Fig. 9:**
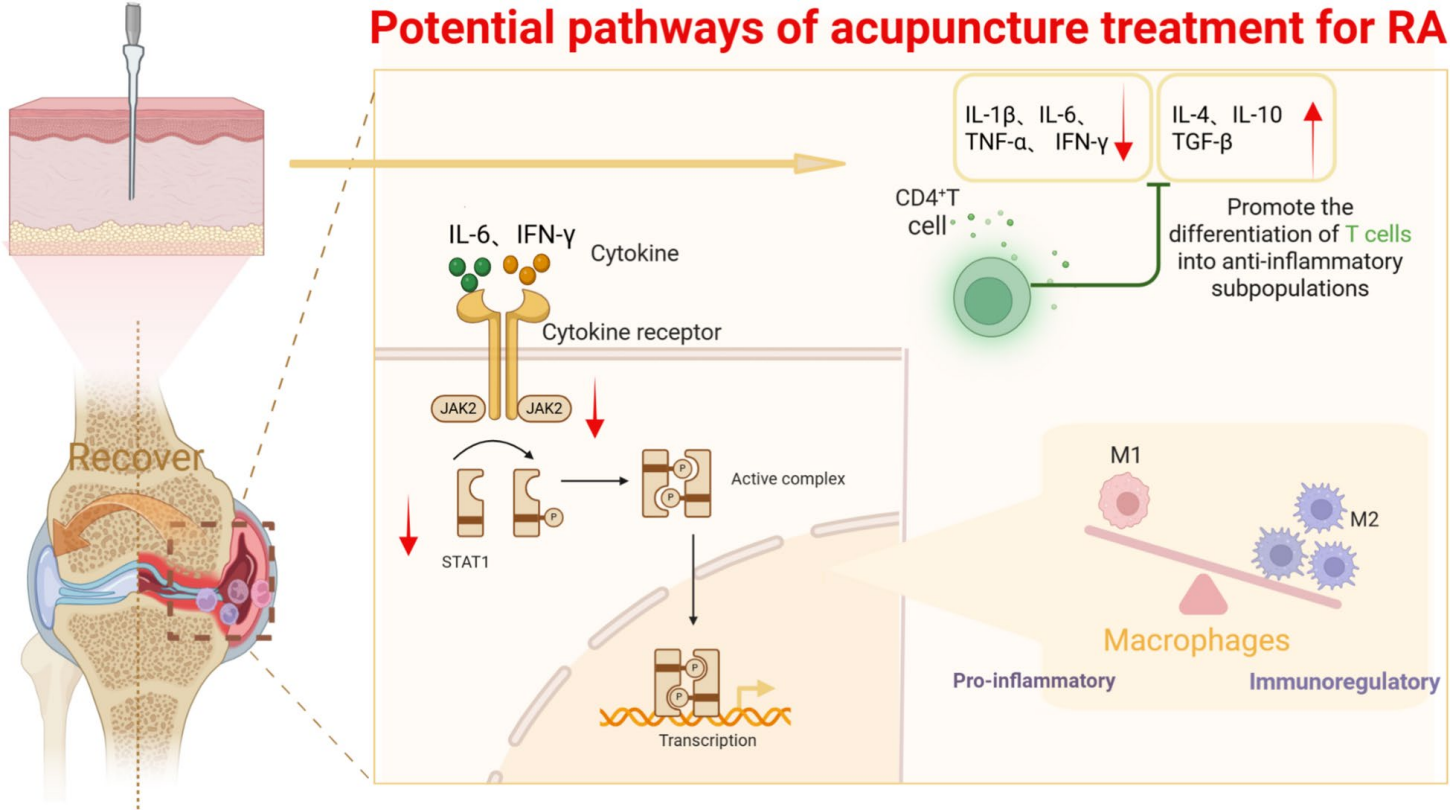
Possible mechanism by which STAT1 is involved in the anti-inflammatory effects of acupuncture

These key DEGs exert anti-inflammatory effects in RA by participating in glycolysis, cellular signaling, and the regulation of cytokine expression and influencing cell survival and apoptosis. Additionally, MR analysis of these five genes revealed a potential causal relationship between elevated levels of STAT1 and increased risk of RA. Acupuncture can provide valuable insights into the pathogenesis of RA by targeting these genes. The results of the present study led us to propose the potential pathways of acupuncture treatment for RA in Fig. 9.

### Limitations and prospects

On the basis of the findings of this study, subsequent research could focus on the following directions: constructing a new evaluation index system, developing clinical diagnostic biomarkers around core targets, and establishing efficacy evaluation criteria on the basis of changes in metabolite profiles after acupuncture intervention. Exploring integrated treatment strategies that combine TCM and Western medicine, such as the combined application of acupuncture and JAK inhibitors and the regulatory effect of acupuncture on the JAK/STAT pathway, may reduce drug dosages, thereby alleviating gastrointestinal adverse reactions. The synergistic potential of acupuncture with other therapies, such as implementing an “acupuncture + low-dose CORT” regimen, should be explored. This strategy aims to increase the anti-inflammatory efficacy of endogenous CORT through acupuncture. However, this study has certain limitations. First, the study heavily relies on public databases such as GEO and IEU, where differences in sample sources, population backgrounds, and sample processing procedures may introduce batch effects, potentially interfering with differential analysis and subsequent machine learning modeling. Future studies should conduct larger-scale surveys across different populations. Second, despite rigorous sensitivity analyses in MR analysis, horizontal pleiotropy and its introduced bias still exist. Third, we analyzed only ten representative active substances produced by acupuncture treatment and failed to cover all the active substances generated postacupuncture. Further data mining and experimental validation are necessary. Fourth, the current study is solely based on bioinformatics correlation analysis and cannot directly prove the causal role and molecular mechanisms of key targets. Future research will validate the direct regulatory effects of DEGs on immune subpopulations through in vitro cell experiments and animal models, further elucidating the molecular mechanisms of the immunoregulatory effects of acupuncture.

## Conclusion

This study integrates TCM’s holistic philosophy with AI-driven precision medicine to systematically identify key active components, action targets, and biomarkers of acupuncture intervention in RA. Research has revealed that endogenous active components (CCK-8, PGE_2_, CORT, and enkephalin) and DEGs (STAT1, GAPDH, JAK2, PTGS2, and MDM2) constitute the core molecular network of acupuncture treatment for RA. STAT1, GAPDH, JAK2, PTGS2, and MDM2 may be potential targets for acupuncture intervention in RA. STAT1, a key regulatory factor, can control immune homeostasis mediated by M1 macrophages and CD4^+^ T cells. This study proposes that acupuncture may regulate the functions of immune cells such as M1 macrophages and CD4^+^ T cells by inhibiting the JAK2/STAT1 signaling axis, reshaping the RA immune microenvironment and exerting anti-inflammatory effects. The innovation of this study Lies in the construction of a multidimensional target network for the acupuncture treatment of RA. By Literature mining, 10 characteristic active components were screened out, and machine learning and Mendelian randomization were subsequently used to identify 5 core regulatory genes, such as STAT1. Subsequent basic experiments are needed to validate and screen the aforementioned substances and molecules.

## Supplementary Information

Additional file 1.

Additional file 2.

Additional file 3.

Additional file 4.

Additional file 5.

Additional file 6.

Additional file 7.

Additional file 8.

Additional file 9.

Additional file 10.

## Data Availability

The data will be made available upon request.

## Abbreviations

RA: Rheumatoid arthritis
GEO: Gene Expression Omnibus
AI: Artificial intelligence
TCM: Traditional Chinese medicine
DMARDs: Disease-modifying antirheumatic drugs
FLS: Fibroblast-like synoviocytes
ROC: Receiver operating characteristic
AUC: Area under the ROC curve
RF: Random forest
SVM: Support vector machine model
GLM: Generalized linear model
XGB: Extreme gradient boosting
MR: Mendelian randomization
IVW: Inverse variance weighting
GSEA: Gene set enrichment analysis
GO: Gene ontology
PPI: Protein‒protein interaction
MCODE: Molecular complex detection
KEGG: Kyoto Encyclopedia of Genes and Genomes
NE: Norepinephrine
CTD: Comparative toxicogenomics database
MENK: Methionine-enkephalin
CCK-8: Cholecystokinin-8
5-HIAA: 5-Hydroxyindole-3-acetic acid
5-HT: Serotonin
DEGs: Differentially expressed genes
COX-2: Cycloxygenase 2
ACTH: AdrenoCorticoTropic hormone
AhR: Aryl hydrocarbon receptor
Bregs: Regulatory B cells
MMPs: Matrix metalloproteinases
Tregs: Regulatory T cells
NK: Natural killer
DCs: Dendritic cells
Th: Helper T cells
HPA: Hypothalamic‒pituitary‒adrenal
JAK: Janus kinase
MENK: Methionine-enkephalin
CORT: Corticosterone
PGE_2_: Prostaglandin E 2
AhR: Aryl hydrocarbon receptor
TSV: Tab-separated values
PCA: Principal component analysis
MF: Molecular function
BP: Biological process
CC: Cellular component
SNPs: Single nucleotide polymorphisms
MMPs: Matrix metalloproteinases

## References

[1] Collaborators GO. Global, regional, and national burden of osteoarthritis, 1990–2020 and projections to 2050: a systematic analysis for the Global Burden of Disease Study 2021. Lancet Rheumatol. 2023;5(9):e508–22. 37675071 10.1016/S2665-9913(23)00163-7 PMC10477960

[2] Farhat H, et al. Increased risk of cardiovascular diseases in rheumatoid arthritis: a systematic review. Cureus. 2022;14(12):e32308. 36632250 10.7759/cureus.32308 PMC9827945

[3] Sokka T, et al. Work disability remains a major problem in rheumatoid arthritis in the 2000s: data from 32 countries in the QUEST-RA study. Arthritis Res Ther. 2010;12(2):R42. 20226018 10.1186/ar2951 PMC2888189

[4] Chung IM, Ketharnathan S, Thiruvengadam M, Rajakumar G. Rheumatoid arthritis: the stride from research to clinical practice. Int J Mol Sci. 2016. 10.3390/ijms17060900. 27338350 10.3390/ijms17060900 PMC4926434

[5] Mazaud C, Fardet L. Relative risk of and determinants for adverse events of methotrexate prescribed at a low dose: a systematic review and meta-analysis of randomized placebo-controlled trials. Br J Dermatol. 2017;177(4):978–86. 28182264 10.1111/bjd.15377

[6] Kirwan JR. Glucocorticoid resistance in patients with rheumatoid arthritis. Scand J Rheumatol. 2007;36(3):165–6. 17657667 10.1080/03009740701340263

[7] Ling S, Jamali F. The effect of infliximab on hepatic cytochrome P450 and pharmacokinetics of verapamil in rats with preadjuvant arthritis: a drug-disease and drug-drug interaction. Basic Clin Pharmacol Toxicol. 2009;105(1):24–9. 19371259 10.1111/j.1742-7843.2009.00405.x

[8] González CM, et al. Perceptions of patients with rheumatic diseases on the impact on daily life and satisfaction with their medications: RHEU-LIFE, a survey to patients treated with subcutaneous biological products. Patient Prefer Adher. 2017;11:1243–52. 10.2147/PPA.S137052 PMC5530861 28790806

[9] Sunil D, Kamath PR. Multi-target directed indole based hybrid molecules in cancer therapy: an up-to-date evidence-based review. Curr Top Med Chem. 2017;17(9):959–85. 27697057 10.2174/1568026616666160927150839

[10] Li N, et al. The anti-inflammatory actions and mechanisms of acupuncture from acupoint to target organs via neuro-immune regulation. J Inflamm Res. 2021;14:14(7191–224. 34992414 10.2147/JIR.S341581 PMC8710088

[11] Wang Y, et al. Effect of moxibustion on β-EP and dyn levels of pain-related indicators in patients with rheumatoid arthritis. Evid Based Complement Alternat Med. 2021;2021:2021(6637554. 33884025 10.1155/2021/6637554 PMC8041546

[12] Yang F, et al. ST36 acupuncture alleviates the inflammation of adjuvant-induced arthritic rats by targeting monocyte/macrophage modulation. Evid Based Complement Alternat Med. 2021;2021:9430501. 33727948 10.1155/2021/9430501 PMC7936911

[13] Wooller SK, Benstead-Hume G, Chen X, Ali Y, Pearl FMG. Bioinformatics in translational drug discovery. 2017. Biosci Rep. 10.1042/BSR20160180. 10.1042/BSR20160180 PMC6448364 28487472

[14] Ye H, Wei J, Tang K, Feuers R, Hong H. Drug repositioning through network pharmacology. Curr Top Med Chem. 2016;16(30):3646–56. 27334200 10.2174/1568026616666160530181328

[15] Boezio B, Audouze K, Ducrot P, Taboureau OJMi. Network-based approaches in pharmacology. Mol Inform. 2017;36(10):1700048. 10.1002/minf.201700048 28692140

[16] Fan AY. Anti-inflammatory mechanism of electroacupuncture involves the modulation of multiple systems, levels and targets and is not limited to “driving the vagus-adrenal axis.” J Integr Med. 2023;21(4):320–3. 37331861 10.1016/j.joim.2023.06.001

[17] Han Z, Zhang Y, Wang P, Tang Q, Zhang K. Is acupuncture effective in the treatment of COVID-19 related symptoms? Based on bioinformatics/network topology strategy. Brief Bioinform. 2021. 10.1093/bib/bbab110. 33866350 10.1093/bib/bbab110 PMC8083275

[18] Wang X, et al. Pharmmapper 2017 update: a web server for potential drug target identification with a comprehensive target pharmacophore database. Nucleic Acids Res. 2017;45(W1):W356–60. 28472422 10.1093/nar/gkx374 PMC5793840

[19] Daina A, Michielin O, Zoete V. SwissTargetPrediction: updated data and new features for efficient prediction of protein targets of small molecules. Nucleic Acids Res. 2019;47(W1):W357–64. 31106366 10.1093/nar/gkz382 PMC6602486

[20] Safran M, et al. The genecards suite. In: Practical guide to life science databases. 2021, p. 27–56.

[21] Wyatt B, et al. Transforming environmental health datasets from the comparative toxicogenomics database into chord diagrams to visualize molecular mechanisms. Front Toxicol. 2024;6:1437884. 39104826 10.3389/ftox.2024.1437884 PMC11298510

[22] Szklarczyk D, et al. The STRING database in 2021: customizable protein-protein networks, and functional characterization of user-uploaded gene/measurement sets. Nucleic Acids Res. 2021;49(D1):D605–12. 33237311 10.1093/nar/gkaa1074 PMC7779004

[23] Rigatti SJ. Random forest. J Insur Med. 2017;47(1):31–9. 28836909 10.17849/insm-47-01-31-39.1

[24] Gold C, Sollich P. Model selection for support vector machine classification. Neurocomputing. 2003;55(1):221–49.

[25] Nelder JA, Wedderburn RWM. Generalized linear models. J R Stat Soc Ser A (General). 1972;135(3):370–84.

[26] Chen T. Xgboost: extreme gradient boosting. package version 2015;1(4):0.4–2.

[27] Higgins JP, Thompson SG, Deeks JJ, Altman DG. Measuring inconsistency in meta-analyses. BMJ. 2003;327(7414):557–60. 12958120 10.1136/bmj.327.7414.557 PMC192859

[28] Burgess S, Thompson SG. Interpreting findings from Mendelian randomization using the MR-Egger method. Eur J Epidemiol. 2017;32(5):377–89. 28527048 10.1007/s10654-017-0255-x PMC5506233

[29] Zhang B, Wu Q, Li B, Wang D, Wang L, Zhou YL. M(6)A regulator-mediated methylation patterns and tumor microenvironment infiltration characterization in gastric cancer. Mol Cancer. 2020;19(1):53. 32164750 10.1186/s12943-020-01170-0 PMC7066851

[30] Giambartolomei C, et al. Bayesian test for colocalisation between pairs of genetic association studies using summary statistics. PLoS Genet. 2014;10(5):e1004383. 24830394 10.1371/journal.pgen.1004383 PMC4022491

[31] McInnes IB, Schett G. The pathogenesis of rheumatoid arthritis. N Engl J Med. 2011;365(23):2205–19. 22150039 10.1056/NEJMra1004965

[32] Zhang Y, et al. Pathological pathway analysis in an experimental rheumatoid arthritis model and the tissue repair effect of acupuncture at ST36. Front Immunol. 2023;14:1164157. 37256145 10.3389/fimmu.2023.1164157 PMC10225595

[33] Wang J, et al. Therapeutic effect and mechanism of acupuncture in autoimmune diseases. Am J Chin Med. 2022;50(03):639–52. 35282807 10.1142/S0192415X22500252

[34] Jang S, Kwon E-J, Lee JJ. Rheumatoid arthritis: pathogenic roles of diverse immune cells. Int J Mol Sci. 2022;23(2):905. 35055087 10.3390/ijms23020905 PMC8780115

[35] Huang Y, et al. Identification of diagnostic genes and drug prediction in metabolic syndrome-associated rheumatoid arthritis by integrated bioinformatics analysis, machine learning, and molecular docking. Front Immunol. 2024;15:1431452. 39139563 10.3389/fimmu.2024.1431452 PMC11320606

[36] Zhou J, et al. Identification of aging-related biomarkers and immune infiltration characteristics in osteoarthritis based on bioinformatics analysis and machine learning. Front Immunol. 2023;14:1168780. 37503333 10.3389/fimmu.2023.1168780 PMC10368975

[37] Du B, Zhu M, Li Y, Li G, Xi X. The prostaglandin E2 increases the production of IL-17 and the expression of costimulatory molecules on γδ T cells in rheumatoid arthritis. Scand J Immunol. 2020;91(5):e12872. 32048307 10.1111/sji.12872

[38] Zhang R, Lao L, Ren K, Berman BM. Mechanisms of acupuncture–electroacupuncture on persistent pain. Anesthesiology. 2014;120(2):482–503. 24322588 10.1097/ALN.0000000000000101 PMC3947586

[39] Zhang R-X, et al. Electroacupuncture attenuates inflammation in a rat model. J Altern Complement Med. 2005;11(1):135–42. 15750372 10.1089/acm.2005.11.135

[40] Wei Y, et al. Regulation of hypothalamic-pituitary-adrenal axis activity and immunologic function contributed to the anti-inflammatory effect of acupuncture in the OVA-induced murine asthma model. Neurosci Lett. 2017;636:177–83. 27816549 10.1016/j.neulet.2016.11.001

[41] Chang FC, Tsai HY, Yu MC, Yi PL, Lin JG. The central serotonergic system mediates the analgesic effect of electroacupuncture on ZUSANLI (ST36) acupoints. J Biomed Sci. 2004;11(2):179–85. 14966368 10.1007/BF02256561

[42] Wang M, Liu W, Ge J, Liu S. The immunomodulatory mechanisms for acupuncture practice. Front Immunol. 2023;14:1147718. 37090714 10.3389/fimmu.2023.1147718 PMC10117649

[43] Ying Z-H, Mao C-L, Xie W, Yu C-H. Postbiotics in rheumatoid arthritis: emerging mechanisms and intervention perspectives. Front Microbiol. 2023;14:1290015. 38029106 10.3389/fmicb.2023.1290015 PMC10662086

[44] Yang Y, et al. The efficacy and neural mechanism of acupuncture therapy in the treatment of visceral hypersensitivity in irritable bowel syndrome. Front Neurosci. 2023;17:1251470. 37732301 10.3389/fnins.2023.1251470 PMC10507180

[45] Wang S, Hong J, Lai X. Influence of electroacupuncture on spinal monoamine neurotransmitter content in rats with adjuvant arthritis. J Guangzhou Univ Tradit Chin Med. 2001;01:1–2+1.

[46] Wang S, Hong J, Zhou Y, Lai X. The influence of electro-acupuncture on Jiaji points on the content of Spinal Cords’ Monoamine neurotransmitter arthritis rats induced by adjuvant. J Guangzhou Univ Tradit. 1999;04:286–8 **(in chinese)**.

[47] de la Fuente M, Medina S, Del Rio M, Ferrández MD, Hernanz A. Effect of aging on the modulation of macrophage functions by neuropeptides. Life Sci. 2000;67(17):2125–35. 11057762 10.1016/s0024-3205(00)00799-2

[48] Trejter M, Warchol J, de Caro R, Brelinska R, Nussdorfer G, Malendowicz L. Studies on the involvement Histology and Histopathology Cellular and Molecular Biology of endogenous neuropeptides in the control of thymocyte proliferation in the rat. Histol Histopathol. 2001;16(1):155–8. 11193190 10.14670/HH-16.155

[49] Lee E-G, Lee S-I, Chae H-J, Park SJ, Lee YC, Yoo W-H. Adrenomedullin inhibits IL-1β-induced rheumatoid synovial fibroblast proliferation and MMPs, COX-2 and PGE2 production. Inflammation. 2011;34:335–43. 20697789 10.1007/s10753-010-9239-7

[50] Zhou Y, Sun Y-H, Shen J-M, Han J-S. Increased release of immunoreactive CCK-8 by electroacupuncture and enhancement of electroacupuncture analgesia by CCK-B antagonist in rat spinal cord. Neuropeptides. 1993;24(3):139–44. 8474632 10.1016/0143-4179(93)90077-n

[51] Han J, Ding X, Fan S. Cholecystokinin octapeptide (CCK-8): antagonism to electroacupuncture analgesia and a possible role in electroacupuncture tolerance. Pain. 1986;27(1):101–15. 3491355 10.1016/0304-3959(86)90227-7

[52] Jie W, et al. Analgesic effect of buccal acupuncture on acute arthritis in rabbits and underlying mechanisms. J Cent South Univ. 2017;42(5):517–21. 10.11817/j.issn.1672-7347.2017.05.006 28626096

[53] Zhao Z. Neural mechanism underlying acupuncture analgesia. Prog Neurobiol. 2008;85(4):355–75. 18582529 10.1016/j.pneurobio.2008.05.004

[54] Cheng L-L, Ding M-X, Xiong C, Zhou M-Y, Qiu Z-Y, Wang Q. Effects of electroacupuncture of different frequencies on the release profile of endogenous opioid peptides in the central nerve system of goats. Evid Based Complement Altern Med Rev. 2012;2012(1):476457. 10.1155/2012/476457 PMC3486625 23133494

[55] Zheng X, Lin J, Wang Z, Zeng Z, Chen H. Research of the analgesic effects and central nervous system impact of electroacupuncture therapy in rats with knee osteoarthritis. Prog Neurobiol. 2024. 10.1016/j.heliyon.2023.e21825. 38226224 10.1016/j.heliyon.2023.e21825 PMC10788782

[56] Fang J, et al. Involvement of peripheral beta-endorphin and MU, delta, kappa opioid receptors in electro acupuncture analgesia for prolonged inflammatory pain of rats. Eur J Inflamm. 2013;11(2):375–83.

[57] Liu J, Dong S, Liu S. Aberrant parasympathetic responses in acupuncture therapy for restoring immune homeostasis. Acupunct Herb Med. 2023;3(2):69–75.

[58] Chen W, et al. Electroacupuncture activated local sympathetic noradrenergic signaling to relieve synovitis and referred pain behaviors in knee osteoarthritis rats. Front Mol Neurosci. 2023;16:1069965. 36959872 10.3389/fnmol.2023.1069965 PMC10028095

[59] Torres-Rosas R, et al. Dopamine mediates vagal modulation of the immune system by electroacupuncture. Nat Med. 2014;20(3):291–5. 24562381 10.1038/nm.3479 PMC3949155

[60] Yan Y, et al. Dopamine controls systemic inflammation through inhibition of NLRP3 inflammasome. Cell. 2015;160(1):62–73. 25594175 10.1016/j.cell.2014.11.047

[61] Yang J, Song K, Liang F, Zhao C, Yang J. The effect of electroacupuncture on the content of inflammatory mediators in the inflamed area of adjuvant arthritis rats. J Chengdu Univ Tradit Chin Med. 1999;01:48–9.

[62] Yuan G. Acupuncture inhibiting the activation of NF-kB through BDNF-TrkB signaling pathway in spinal cord to ameliorate complete Freund's adjuvant-induced inflammatory pain of mice [master]. Tianjin University of Traditional Chinese Medicine; 2021.

[63] Mai J, Lu M, Gao Q, Zeng J, Xiao J. Transcriptome-wide association studies: recent advances in methods, applications and available databases. Commun Biol. 2023;6(1):899. 37658226 10.1038/s42003-023-05279-y PMC10474133

[64] Zhang JG, Liu JX, Jia XX, Geng J, Yu F, Cong B. Cholecystokinin octapeptide regulates the differentiation and effector cytokine production of CD4(+) T cells *in vitro*. Int Immunopharmacol. 2014;20(2):307–15. 24704498 10.1016/j.intimp.2014.03.013

[65] Crawley JN, Corwin RL. Biological actions of cholecystokinin. Peptides. 1994;15(4):731–55. 7937354 10.1016/0196-9781(94)90104-x

[66] Zhang JG, et al. Cholecystokinin octapeptide inhibits immunoglobulin G1 production of lipopolysaccharide-activated B cells. Int Immunopharmacol. 2011;11(11):1685–90. 21664492 10.1016/j.intimp.2011.05.027

[67] Li Q, et al. Cholecystokinin octapeptide significantly suppresses collagen-induced arthritis in mice by inhibiting Th17 polarization primed by dendritic cells. Cell Immunol. 2011;272(1):53–60. 22004797 10.1016/j.cellimm.2011.09.007

[68] Xia X, et al. Single cell immunoprofile of synovial fluid in rheumatoid arthritis with TNF/JAK inhibitor treatment. Nat Commun. 2025;16(1):2152. 40038288 10.1038/s41467-025-57361-0 PMC11880340

[69] Tang CW, Feng WM, Du HM, Bao Y, Zhu M. Delayed administration of D-Ala2-D-Leu5-enkephalin, a delta-opioid receptor agonist, improves survival in a rat model of sepsis. Tohoku J Exp Med. 2011;224(1):69–76. 21551984 10.1620/tjem.224.69

[70] Xing J, Xia M, Wang T, Mu JA. Study on the analgesic effect of acupuncture with opioid receptors agonist in induced arthritic rats. Acupunct Res. 1989;14(3):375–8. 2512029

[71] Tsuge K, Inazumi T, Shimamoto A, Sugimoto Y. Molecular mechanisms underlying prostaglandin E2-exacerbated inflammation and immune diseases. Int Immunol. 2019;31(9):597–606. 30926983 10.1093/intimm/dxz021

[72] Sreeramkumar V, Fresno M, Cuesta N. Prostaglandin E2 and T cells: friends or foes? Immunol Cell Biol. 2012;90(6):579–86. 21946663 10.1038/icb.2011.75 PMC3389798

[73] Jianzhen J, et al. Efficacy of electroacupuncture stimulating Zusanli (ST36) and Xuanzhong (GB39) on synovial angiogenesis in rats with adjuvant arthritis. J Tradit Chin Med. 2023;43(5):955–62. 37679983 10.19852/j.cnki.jtcm.20221111.002 PMC10465822

[74] Oufkir T, Vaillancourt C. Phosphorylation of JAK2 by serotonin 5-HT (2A) receptor activates both STAT3 and ERK1/2 pathways and increases growth of JEG-3 human placental choriocarcinoma cell. Placenta. 2011;32(12):1033–40. 21993263 10.1016/j.placenta.2011.09.005

[75] Sahu A, et al. The 5-hydroxytryptamine signaling map: an overview of serotonin-serotonin receptor mediated signaling network. J Cell Commun Signal. 2018;12(4):731–5. 30043327 10.1007/s12079-018-0482-2 PMC6235773

[76] Morales JK, Falanga YT, Depcrynski A, Fernando J, Ryan JJ. Mast cell homeostasis and the JAK-STAT pathway. Genes Immun. 2010;11(8):599–608. 20535135 10.1038/gene.2010.35 PMC3099592

[77] Hamidzadeh K, Christensen SM, Dalby E, Chandrasekaran P, Mosser DM. Macrophages and the recovery from acute and chronic inflammation. Annu Rev Physiol. 2017;79:567–92. 27959619 10.1146/annurev-physiol-022516-034348 PMC5912892

[78] Feng Y, Lu Y. Immunomodulatory effects of dopamine in inflammatory diseases. Front Immunol. 2021;12:663102. 33897712 10.3389/fimmu.2021.663102 PMC8063048

[79] Fukuhara Y, Takeshima T, Kashiwaya Y, Shimoda K, Ishitani R, Nakashima K. GAPDH knockdown rescues mesencephalic dopaminergic neurons from MPP+ -induced apoptosis. NeuroReport. 2001;12(9):2049–52. 11435944 10.1097/00001756-200107030-00051

[80] Bellinger DL, Wood C, Wergedal JE, Lorton D. Driving β(2)- while suppressing α-adrenergic receptor activity suppresses joint pathology in inflammatory arthritis. Front Immunol. 2021;12:628065. 34220796 10.3389/fimmu.2021.628065 PMC8249812

[81] Straub RH, Dufner B, Rauch L. Proinflammatory α-adrenergic neuronal regulation of splenic IFN-γ, IL-6, and TGF-β of mice from day 15 onward in arthritis. NeuroImmunoModulation. 2020;27(1):58–68. 32610310 10.1159/000508109 PMC7446300

[82] Ishii Y, et al. Anti-inflammatory effects of noradrenaline on LPS-treated microglia: suppression of NFκB nuclear translocation and subsequent STAT1 phosphorylation. Neurochem Int. 2015;90:56–66. 26190182 10.1016/j.neuint.2015.07.010

[83] Dickmeis T, Foulkes NS. Glucocorticoids and circadian clock control of cell proliferation: at the interface between three dynamic systems. Mol Cell Endocrinol. 2011;331(1):11–22. 20833224 10.1016/j.mce.2010.09.001

[84] Ao Y, Wang Z, Hu J, Yao M, Zhang W. Identification of essential genes and immune cell infiltration in rheumatoid arthritis by bioinformatics analysis. Sci Rep. 2023;13(1):2032. 36739468 10.1038/s41598-023-29153-3 PMC9899220

[85] Zhou S, Lu H, Xiong M. Identifying immune cell infiltration and effective diagnostic biomarkers in rheumatoid arthritis by bioinformatics analysis. Front Immunol. 2021;12:726747. 34484236 10.3389/fimmu.2021.726747 PMC8411707

[86] Boutet M-A, et al. Novel insights into macrophage diversity in rheumatoid arthritis synovium. Autoimmun Rev. 2021;20(3):102758. 33476818 10.1016/j.autrev.2021.102758

[87] Gao Y, Cai W, Zhou Y, Li Y, Cheng J, Wei F. Immunosenescence of T cells: a key player in rheumatoid arthritis. Inflamm Res. 2022;71(12):1449–62. 36280621 10.1007/s00011-022-01649-0

[88] Tang M, Tian L, Luo G, Yu X. Interferon-gamma-mediated osteoimmunology. Front Immunol. 2018;9:1508. 30008722 10.3389/fimmu.2018.01508 PMC6033972

[89] Roberts CA, Dickinson AK, Taams LS. The interplay between monocytes/macrophages and CD4+ T-cell subsets in rheumatoid arthritis. Front Immunol. 2015;6:571. 26635790 10.3389/fimmu.2015.00571 PMC4652039

[90] Kondo Y, et al. Transcriptional regulation of CD 4+ T-cell differentiation in experimentally induced arthritis and rheumatoid arthritis. Arthritis Rheumatol. 2018;70(5):653–61. 29245178 10.1002/art.40398 PMC5947164

[91] Sun W, et al. B cells inhibit bone formation in rheumatoid arthritis by suppressing osteoblast differentiation. Nat Commun. 2018;9(1):5127. 30510188 10.1038/s41467-018-07626-8 PMC6277442

[92] Wu F, et al. B cells in rheumatoid arthritis: pathogenic mechanisms and treatment prospects. Front Immunol. 2021;12:750753. 34650569 10.3389/fimmu.2021.750753 PMC8505880

[93] Lei Y, et al. Synovial microenvironment-influenced mast cells promote the progression of rheumatoid arthritis. Nat Commun. 2024;15(1):113. 38168103 10.1038/s41467-023-44304-w PMC10761862

[94] Kucuksezer UC, et al. The role of natural killer cells in autoimmune diseases. Front Immunol. 2021;12:622306. 33717125 10.3389/fimmu.2021.622306 PMC7947192

[95] Oh JE, Kim SN. Anti-inflammatory effects of acupuncture at ST36 point: a literature review in animal studies. Front Immunol. 2021;12:813748. 35095910 10.3389/fimmu.2021.813748 PMC8790576

[96] Loucks A, Maerz T, Hankenson K, Moeser A, Colbath A. The multifaceted role of mast cells in joint inflammation and arthritis. Osteoarthr Cartil. 2023;31(5):567–75. 10.1016/j.joca.2023.01.005 36682447

[97] Kasperkovitz PV, et al. Activation of the STAT1 pathway in rheumatoid arthritis. Ann Rheum Dis. 2004;63(3):233–9. 14962955 10.1136/ard.2003.013276 PMC1754903

[98] Monari C, et al. A microbial polysaccharide reduces the severity of rheumatoid arthritis by influencing Th17 differentiation and proinflammatory cytokines production. J Immunol. 2009;183(1):191–200. 19542430 10.4049/jimmunol.0804144

[99] Ciobanu DA, et al. JAK/STAT pathway in pathology of rheumatoid arthritis (Review). Exp Therap Med. 2020;20(4):3498–503. 32905201 10.3892/etm.2020.8982 PMC7465448

[100] Owen KL, Brockwell NK, Parker BS. Jak-stat signaling: a double-edged sword of immune regulation and cancer progression. Cancers. 2019. 10.3390/cancers11122002. 31842362 10.3390/cancers11122002 PMC6966445

[101] Liang YB, et al. Downregulated SOCS1 expression activates the JAK1/STAT1 pathway and promotes polarization of macrophages into M1 type. Mol Med Rep. 2017;16(5):6405–11. 28901399 10.3892/mmr.2017.7384

[102] Elbrashy MM, Metwally H, Sakakibara S, Kishimoto T. Threonine phosphorylation and the yin and yang of STAT1: phosphorylation-dependent spectrum of STAT1 functionality in inflammatory contexts. Cells. 2024. 10.3390/cells13181531. 10.3390/cells13181531 PMC11429647 39329714

[103] Zhang M, Xu M, Wang K, Li L, Zhao J. Effect of inhibition of the JAK2/STAT3 signaling pathway on the Th17/IL-17 axis in acute cellular rejection after heart transplantation in mice. J Cardiovasc Pharmacol. 2021;77(5):614–20. 33951698 10.1097/FJC.0000000000001007 PMC8096315

[104] Lv Y, et al. The JAK-STAT pathway: from structural biology to cytokine engineering. Signal Transduct Target Ther. 2024;9(1):221. 39169031 10.1038/s41392-024-01934-w PMC11339341

[105] Yang X, et al. Cell volume regulation modulates macrophage-related inflammatory responses via JAK/STAT signaling pathways. Acta Biomater. 2024;186:286–99. 39098445 10.1016/j.actbio.2024.07.046

[106] Sarapultsev A, Gusev E, Komelkova M, Utepova I, Luo S, Hu D. Jak-STAT signaling in inflammation and stress-related diseases: implications for therapeutic interventions. Mol Biomed. 2023;4(1):40. 37938494 10.1186/s43556-023-00151-1 PMC10632324

[107] Fan X, et al. Comprehensive landscape-style investigation of the molecular mechanism of acupuncture at ST36 single acupoint on different systemic diseases. Heliyon. 2024;10(4):e26270. 38375243 10.1016/j.heliyon.2024.e26270 PMC10875596

[108] Muñoz A, Costa M. Nutritionally mediated oxidative stress and inflammation. Oxid Med Cell Longev. 2013;2013:610950. 23844276 10.1155/2013/610950 PMC3697417

[109] Chen Y, et al. PTGS2: A potential immune regulator and therapeutic target for chronic spontaneous urticaria. Life Sci. 2024;344:122582. 38514006 10.1016/j.lfs.2024.122582

[110] Martín-Vázquez E, Cobo-Vuilleumier N, López-Noriega L, Lorenzo PI, Gauthier BR. The PTGS2/COX2-PGE(2) signaling cascade in inflammation: pro or anti? A case study with type 1 diabetes mellitus. Int J Biol Sci. 2023;19(13):4157–65. 37705740 10.7150/ijbs.86492 PMC10496497

[111] Butterfield DA, Hardas SS, Lange ML. Oxidatively modified glyceraldehyde-3-phosphate dehydrogenase (GAPDH) and Alzheimer’s disease: many pathways to neurodegeneration. J Alzheimers Dis. 2010;20(2):369–93. 20164570 10.3233/JAD-2010-1375 PMC2922983

[112] Soto-Heredero G, Gómez de Las Heras MM, Gabandé-Rodríguez E, Oller J, Mittelbrunn M. Glycolysis—a key player in the inflammatory response. FEBS J. 2020;287(16):3350–69. 32255251 10.1111/febs.15327 PMC7496292

[113] Yu Q, et al. Interactions between NLRP3 inflammasome and glycolysis in macrophages: new insights into chronic inflammation pathogenesis. Immun Inflamm Dis. 2022;10(3):e581. 34904398 10.1002/iid3.581 PMC8926505

[114] Rao Y, et al. Delivery of long noncoding RNA NEAT1 by peripheral blood monouclear cells-derived exosomes promotes the occurrence of rheumatoid arthritis via the microRNA-23a/MDM2/SIRT6 Axis. Front Cell Dev Biol. 2020;8:551681. 33042992 10.3389/fcell.2020.551681 PMC7517357

[115] Thomasova D, Mulay SR, Bruns H, Anders HJ. P53-independent roles of MDM2 in NF-κB signaling: implications for cancer therapy, wound healing, and autoimmune diseases. Neoplasia. 2012;14(12):1097–101. 23308042 10.1593/neo.121534 PMC3540936

[116] Wang W, Xiang T, Yang Y, Wang Z, Xie J. E3 ubiquitin ligases STUB1/CHIP contributes to the Th17/Treg imbalance via the ubiquitination of aryl hydrocarbon receptor in rheumatoid arthritis. Clin Exp Immunol. 2022;209(3):280–90. 35943876 10.1093/cei/uxac072 PMC9521662

[117] Wu KK, et al. MDM2 induces pro-inflammatory and glycolytic responses in M1 macrophages by integrating iNOS-nitric oxide and HIF-1α pathways in mice. Nat Commun. 2024;15(1):8624. 39366973 10.1038/s41467-024-53006-w PMC11452520

