# Supplementary figures and images for: Potential mechanisms of acupuncture treatment for rheumatoid arthritis: a study based on network topology and machine learning
Source: Chin Med. 2025 Oct 7;20:164. doi: 10.1186/s13020-025-01209-8 (PMC12502209; doi:10.1186/s13020-025-01209-8)

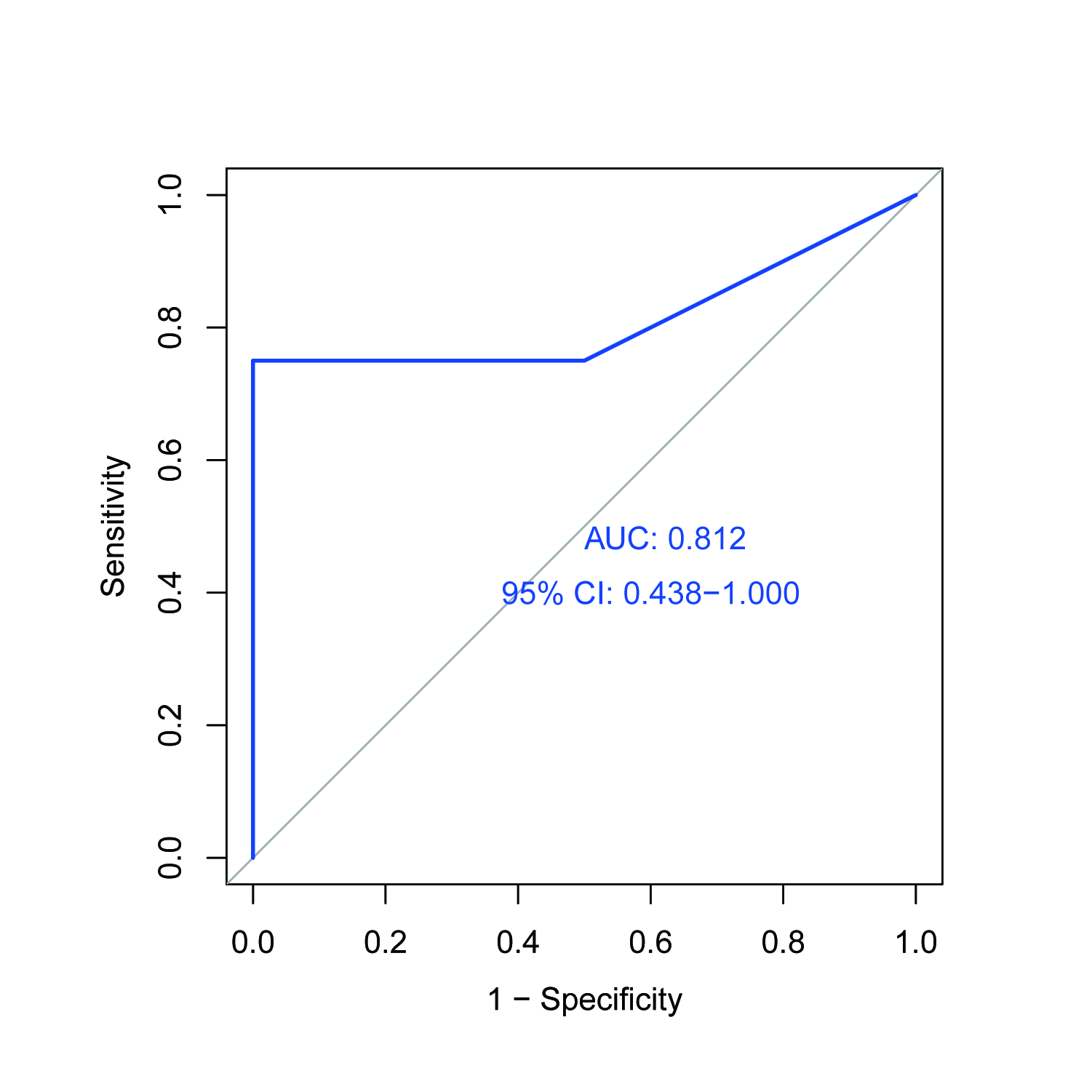

Supplement: Supplementary file 1 — Additional file 1. [file 13020_2025_1209_MOESM1_ESM.jpeg]
