# Supplementary material for: Potential mechanisms of acupuncture treatment for rheumatoid arthritis: a study based on network topology and machine learning
Source: Chin Med. 2025 Oct 7;20:164. doi: 10.1186/s13020-025-01209-8 (PMC12502209; doi:10.1186/s13020-025-01209-8)
Supplement: Supplementary file 3 — Additional file 3. [file 13020_2025_1209_MOESM3_ESM.docx]

**Acupuncture treatment for rheumatoid arthritis-related compounds**

(Bioactive components involved in neuroregulation, immune modulation, and inflammatory response, which align with the “holistic regulation” characteristic of acupuncture)

| **Refs** | **Year** | **Author** | **Journal** | **Title** | **Article Type** | **Bioactive components** |
| --- | --- | --- | --- | --- | --- | --- |
| [1] | 2013 | Y Shou | Evidence-Based Complementary and Alternative Medicine | Electroacupuncture inhibition of hyperalgesia in rats with adjuvant arthritis: involvement of cannabinoid receptor 1 and dopamine receptor subtypes in striatum | basic research | Dopamine |
| [2] | 2011 | YC Yoo | Yonsei Med J | Analgesic mechanism of electroacupuncture in an arthritic pain model of rats: a neurotransmitter study. | basic research | Epinephrine  Serotonin  Dopamine |
| [3] | 2012 | M Huang | [Evidence-Based Complementary and Alternative Medicine](https://onlinelibrary.wiley.com/journal/4747) | In adjuvant-induced arthritic rats, acupuncture analgesic effects are histamine dependent: potential reasons for acupoint preference in clinical practice. Evid. Based Complement | Clinical research | Histamine |
| [4] | 2016 | BK Seo | [BMC Complementary and Alternative Medicine](https://link.springer.com/journal/12906) | The electroacupuncture-induced analgesic effect mediated by 5-HT1, 5-HT3 receptor and muscarinic cholinergic receptors in rat model of collagenase-induced osteoarthritis. | basic research | Serotonin  5-HIAA |
| [5] | 2018 | XC Yuan | Front. Mol. Neurosci Sec.Pain Mechanisms and Modulators | Electroacupuncture potentiates cannabinoid receptor-mediated descending inhibitory control in a mouse model of knee osteoarthritis | basic research | Serotonin |
| [6] | 2019 | H Zhu | Front. Neurosci. | Inhibition of GABAergic neurons and excitation of glutamatergic neurons in the ventrolateral periaqueductal gray participate in electroacupuncture analgesia mediated by cannabinoid receptor | basic research | GABA Glutamate |
| [7] | 2018 | W Chai | [Evidence-Based Complementary and Alternative Medicine](https://onlinelibrary.wiley.com/journal/4747) | Electroacupuncture alleviates pain responses and inflammation in a rat model of acute gout arthritis | basic research | β-endorphin |
| [8] | 2007 | A Li | [BMC Complementary and Alternative Medicin](https://link.springer.com/journal/12906) | Corticosterone mediateselectroacupuncture-produced anti-edema in a ratmodel of inflammation. | basic research | Corticosterone |
| [9] | 2008 | A Li | [BMC Complementary and Alternative Medicin](https://link.springer.com/journal/12906) | Electroacupuncture activates corticotrophin-releasing hormone-containingneurons in the paraventricular nucleus of thehypothalammus to alleviate edema in a rat modelofinflammation | basic research | Corticosterone  ACTH |
| [10] | 2010 | Yoichi Minakawa | Pain Research | Effect of electroacupuncture on carrageenan-induced muscle pain in rats | basic research | ACTH |
| [11] | 2002 | YQ Zhang | Pain | Excitatoryamino acid receptor antagonists andelectroacupuncture synergetically inhibitcarrageenan-induced behavioral hyperalgesiaand spinal fos expression in rats. | basic research | Serotonin Norepinephrine |
| [12] | 1997 | LX Zhang | Brain research | Rats withdecreased brain cholecystokinin levels showincreased responsiveness to peripheral electricalstimulation-induced analgesia | basic research | CCK-8 |
| [13] | 2003 | GS Lee | Brain research | Enhancement of electroacupuncture-induced analgesic effect in cholecystokinin-Areceptor defcient rats | basic research | CCK-8 |
| [14] | 1997 | NM Tang | Pain | Cholecystokinin antisense RNA increases theanalgesic effect induced by electroacupuncture orlow dose morphine: Conversion of low responderrats into high responders | basic research | CCK-8 |
| [15] | 2007 | C Huang | Brain research bulletin | CCK(B) receptor antagonist L365,260 potentiates theeffcacy to and reverses chronic tolerance toelectroacupuncture-induced analgesia in mice | basic research | CCK-8 |
| [16] | 2013 | Woojin Kim | Evidence-Based Complementary and Alternative Medicine | Mechanisms of Electroacupuncture-Induced Analgesia on Neuropathic Pain in Animal Model | basic research | Dopamine Serotonin  5-HIAA Norepinephrine |
| [17] | 1989 | [H Y Tsai](https://pubmed.ncbi.nlm.nih.gov/?term=Tsai+HY&cauthor_id=2471859) | The Japanese Journal of Pharmacology | Further Evidence for Possible Analgesic Mechanism of Electroacupuncture: Effects on Neuropeptides and Serotonergic Neurons in Rat Spinal Cord | basic research | MET-enkephalin  Serotonin  SP |
| [18] | 2001 | Shengxu Wang | Chinese Journal of Traditional Medical Science and Technology | The Influence of Electro-Acupuncture on Jiaji Points on The Content of Spinal Cords' Monoamine Neurotransmittttter Arihritis Rats Induced By Adjuvant (in Chinese) | basic research | Dopamine Norepinephrine Serotonin  5-HIAA |
| [19] | 1996 | Jianyang Xu | Chinese Acupuncture & Moxibustion | Effect of Electroacupuncture on Expression of C- Fos in Hipocampus in the Rat with Experimental Rheuma- toid Arthritis (in Chinese) | basic research | Serotonin Dopamine Norepinephrine |
| [20] | 1999 | Shengxu Wang | Chinese Journal of Traditional Medical Science and Technology | Effect of Bilateral Paravertebral Electroacupuncture on Spinal Prodynorphin mRNA Expression in Rats with Adjuvant-Induced Arthritis (in Chinese) | basic research | Deltorphin |
| [21] | 2000 | Shengxu Wang | Chinese Journal of Traditional Medical Science and Technology | Immunohistochemical Evaluation of Electroacupuncture at Jiaji Points on Substance P Expression in the Spinal Cord of Rats with Freund’s Adjuvant-Induced Arthritis (in Chinese) | basic research | SP |
| [22] | 2000 | Shengxu Wang | Chinese Journal of Traditional Medical Science and Technology | Effect of Electroacupuncture at Jiaji Points on Peripheral Blood Monoamine Levels in Rats with Adjuvant-Induced Arthritis (in Chinese) | basic research | Serotonin  5-HIAA |
| [23] | 1999 | JinrongYang | Journal of Chengdu University of Traditional Chinese Medicine | The effect of electroacupuncture on the content of inflammatory mediators in the inflamed area of adjuvant arthritis rats. (in Chinese) | basic research | Histamine  Serotonin  5-HIAA  Dinoprostone |
| [24] | 2002 | Dijun Huang | Shanghai Journal of Acupuncture and Moxibustion | Effect of Moxibustion with Grain-Sized Moxa Cone on Local Tissue 5-HT, HA and PGE2 in Experimental RA Rats (in Chinese) | basic research | Histamine  Serotonin  Dinoprostone |
| [25] | 1999 | Canghuan Zhao | Journal of Basic Chinese Medicine | The effect of electroacupuncture on local β-EP and LEK content in adjuvant arthritis rats (in Chinese) | basic research | LEU-enkephalin  β-endorphin |
| [26] | 2015 | Xiuhua Gao | Journal of Basic Chinese Medicine | Effects of Different Moxibustion on the Function of HPAA in Rabbits with Rheumatoid Arthritis (in Chinese) | basic research | CRH  CCK-8  ACTH |
| [27] | 2015 | Wenbin Ma | Acupuncture Research | Effects of Chronological Moxibustion on Circadian Rhythm Activities of Hypothalamus-Pituitary-Axis in Rheumatoid Arthritis Rats (in Chinese) | basic research | CRH  Corticosterone ACTH |
| [28] | 1999 | Jiebin Yang | Chinese Acupuncture & Moxibustion | A study on the peripheral analgesic mechanisms of different acupuncture therapies in adjuvant-induced arthritis rats (in Chinese) | basic research | Dinoprostone  Histamine  5-H-AA Norepinephrine |
|  |  |  |  |  |  | LEU-enkephalin  β-endorphin |
| [29] | 2016 | Xuguang Liu | Journal of Basic Chinese Medicine | The effect of moxibustion on the hypothalamic-pituitary-adrenal axis in rats with rheumatoid arthritis (in Chinese) | basic research | Corticosterone |
| [30] | 2008 | Jiaolu Wei | Journal of Chengdu Medical College | The Difference of Effect on Plasma Corticosterone Content of Adjuvant-induced Arths Rats between Acupuncture and Moxibustion Therapy (in Chinese) | basic research | Corticosterone |
| [31] | 1997 | Xinlian Liu | Shanghai Journal of Acupuncture and oxibustion | The impact of warming on the immune function of patients with rheumatoid arthritis (in Chinese) | Clinical research | LEU-enkephalin |
| [32] | 2015 | Yongliang Jiang | Chinese Acupuncture & Moxibustion | Anti-inflammatory and synovial-opioid system effects of electroacupuncture intervention on chronic pain in arthritic rats (in Chinese) | basic research | MET-enkephalinβ-endorphin |
| [33] | 1987 | Jian Mu | Journal of Nanjing Medical University (Natural) | A study on the analgesic effect of acupuncture on experimental arthritis in rats (in Chinese) | basic research | MET-enkephalin LEU-enkephalin |
| [34] | 1984 | Kiser S | Information on Traditional Chinese Medicine | The relationship between acupuncture for alleviating chronic pain syndrome and increased plasma methionine-enkephalin (MEK) concentration. (in Chinese) | Clinical research | MET-enkephalin |
| [35] | 2016 | Hu Wang | Zhejiang Journal of Traditional Chinese Medicine | A study on the correlation between hypothalamic enkephalin and its degradation enzyme content and the efficacy of electroacupuncture analgesia. (in Chinese) | basic research | MET-enkephalin  LEU-enkephalin |
| [36] | 2021 | Chuanyu Peng China | Journal of Traditional Chinese Medicine and Pharmacy | The effect of acupuncture combined with moxibustion on the expression of TRPV1 in dorsal root ganglia of rats with rheumatoid arthritis (in Chinese) | basic research | SP  CGRP |
| [37] | 2010 | Ling Luo | Chengdu University of Traditional Chinese Medicine | Research on the local mechanism of immune regulation by moxibustion (in Chinese) | basic research | Corticosterone |
| [38] | 2000 | Zhaoliang Tang | Journal of Basic Chinese Medicin | Experimental study on effects of moxibustion's anti inflammatory and immunity action on neurotransmitter (in Chinese) | Clinical research | Norepinephrine Epinephrine |
| [39] | 2021 | Junrong Bian | Chinese Medicine Modern Distance Education of China | Clinical Effect of Huangqi Jianzhong Decoction Combined with Warm Acupuncture on Rheumatoid Arthritis (in Chinese) | Clinical research | Epinephrine |
| [40] | 2019 | Fuyu Wang | Shanghai Journal of Acupuncture and Moxibustion | Observation on the Efficacy of Warm Needling Moxibustion as Mainly in Treating Active Rheumatoid Arthritis (in Chinese) | Clinical research | Epinephrine Norepinephrine Corticosterone Dopamine |
| [41] | 2023 | Yanfei Feng | Clinical Research and Practice | Effect of warm acupuncture combined with modified Taohong Yin in the treatment of rheumatoid arthritis of blood stasis blocking collaterals and its influences on bone metabolism, MCP-1 and TGF-β1(in Chinese) | Clinical research | Histamine |
| [42] | 2017 | Jingjing Li | Journal of Jinan University (Natural Science & Medicine Edition) | Evaluation of the effects of fireneedle on the levels of 5-HT, HA and PGE2 in rheumatoid arthritis rats by microdialysis (in Chinese) | basic research | Histamine  Serotonin  Dinoprostone |
| [43] | **2001** | Yiling Wang | Journal of Zhengzhou University (Medical Sciences) | **Effects of eletroacupuncture on met-enkephalin, dynorphin in patient plasma and mouse spinal cord and analgesia** (in Chinese) | basic research | MET-enkephalin  Deltorphin |
| [44] | **1997** | Zhuhong Ma | Chinese Acupuncture & Moxibustion | **A clinical study on the impact of acupuncture on the immune-neuroendocrine system of patients with rheumatoid arthritis** (in Chinese) | Clinical research | LEU-enkephalin Dinoprostone |
| [45] | 1996 | Fei Luo | Chinese Journal of Pain Medicine | Repeated Electroacupuncture Modulates Spontaneous Release of Substance P, Cholecystokinin-QOctapeptide, and Methionine-Enkephalin in Rats with Adjuvant-Induced Monoarthritis (in Chinese) | basic research | SP  CCK-8 MET-enkephalin |
| [46] | 1994 | Caiyuan Wang | Acta Medicinae Universitatis Scientiae et Technologiae | The Influence of Central and Peripheral Acetylcholineon the Brain Methionine Enkephalin of Rat andTheir Relations with Eletroacupuncture (in Chinese) | basic research | ACH  MET-enkephalin |
| [47] | 2009 | Bo Ji | Journal of Beijing University of Traditional Chinese Medicine | Effects of acupotome dissolution therapy and electro-acupuncture on central monoamine neurotransmitters in rabbits with knee osteoarthritis (in Chinese) | basic research | Serotonin  5-HIAA Norepinephrine  HVA  DOPAC |
| [48] | 1988 | NingYan Qiu | Acupuncture Research | Influence of Analgesics and Acupuncture on Plasma Cyclic Adenosine Monophosphate Content in a Rat Model of Adjuvant-Induced Arthritis (in Chinese) | basic research | MET-enkephalin |
| [49] | 1997 | Xinlian Liu | Academic Journal of Chinese PLA Medical School | The effects of warm acupuncture on the serum L-ENK，IL-2and the activity of NK cell in patients with rheumatoid arthritis. (in Chinese) | Clinical research | LEU-enkephalin |
| [50] | 2010 | Bo Ji | Chinese Journal of Pathophysiology | Effects of acupotome and electro-acupuncture on pain threshold and monoamine-neurotransmitters of central nerves in rats with knee osteoarthritis (in Chinese) | basic research | Serotonin Norepinephrine Dopamine |
| [51] | 2007 | Xuezhi Li | Journal of Practical Traditional Chinese Medicine | Study on Effect and Mechanism of Analgesia on Adjuvant-induced Arthritis Rats with Crossing Selection of Acupoints Method (in Chinese) | basic research | β-endorphin  ACTH  Deltorphin |
| [52] | 2022 | [Qian Tan](https://kns.cnki.net/kcms2/author/detail?v=9IId9Ku_yBYdc98vT7GMkuY-MrT_HHreqGrz61LIGFRuUu_K_-mTxqFH4aQVYsc-5dLRRUM5aano-1c0e2B_eks3HRnv66snZ1OHE-W0llmG65JBznJ3QA==&uniplatform=NZKPT&language=CHS) | World Chinese Medicine | Effect of Acupuncture at *Zusanli* (ST 36) on Gastrointestinal Motility in Rats with Collagen-induced Arthritis (in Chinese) | basic research | VIP |
| [53] | 1997 | Shuying Yin | Acupuncture Research | Effects of Warm Needling on the Concentrations of PlasmaPostaglandin E, in the Patients with Rheumatoid Arthritis (in Chinese) | basic research | Dinoprostone |
| [54] | 2016 | Jing Han | Shanghai Journal of Acupuncture and Moxibustion | Effect of Electroacupuncture on the Expression of Adenosine A1 Receptor in Hypothalamus and Spinal Cord of Adjuvant Arthritis Rats (in Chinese) | basic research | Adenosine |
| [55] | 2001 | Liang Hao | Chengdu University of Traditional Chinese Medicine | The regulatory mechanism of moxibustion on the neuroimmuneendocrine network in experimental RA rats (in Chinese) | basic research | Corticosterone |
| [56] | 2009 | Xiaorong Chang | Chinese Archives of Traditional Chinese Medicine | The Im pact ofE lectroacupunc turng on PontZusanlj Guanyuan toβ -EP, ACTH ofAdjuvantnduced A rhritis Rats (in Chinese) | basic research | [β-endorphin](https://qikan.cqvip.com/Qikan/Search/Index?key=K=%ce%92-%e5%86%85%e5%95%a1%e8%82%bd&from=Qikan_Search_Index)  [ACTH](https://qikan.cqvip.com/Qikan/Search/Index?key=K=ACTH&from=Qikan_Search_Index) |
| [57] | 2005 | Hui Li | Chinese Acupuncture & Moxibustion | Effects of electroacupuncture on CRH, IL-2 and β-EP contents in the hypothalamus in the rat of adjuvant arthritis (in Chinese) | basic research | [β-endorphin](https://qikan.cqvip.com/Qikan/Search/Index?key=K=%ce%92-%e5%86%85%e5%95%a1%e8%82%bd&from=Qikan_Search_Index)  CRH |
| [58] | 2012 | Chaozhan Ren | Chinese Journal of Information on Traditional Chinese Medicine | Analgesic Effect of Buccal Acupuncture and Changes of β-EP and CCK-8 in Cerebrospinal Fluid of Experimental Rheumatoid Arthritis Rabbits (in Chinese) | basic research | CCK-8  β-endorphin |
| [59] | 2017 | Wangjun Jie | Journal of Central South University (Medical Science) | Analgesic effect of buccal acupuncture on acute arthritis in rabbits and underlying mechanisms (in Chinese) | basic research | [β-endorphin](https://qikan.cqvip.com/Qikan/Search/Index?key=K=%ce%92-%e5%86%85%e5%95%a1%e8%82%bd&from=Qikan_Search_Index)  CCK-8 |
| [60] | 2003 | Manying Xu | Chinese Journal of Tissue Engineering Research | A study on the mechanism of how the levels of cholecystokinin octapeptide in the brain affect the efficacy of acupuncture analgesia. (in Chinese) | basic research | CCK-8 |
| [61] | 1998 | Yong Huang and Technology | Chinese Journal of Traditional Medical Science | Observation of circadian rhythms of plasma dopamine in adjuvant arthritis rabbits and the effect of acupuncture on it (in Chinese) | basic research | Dopamine |
| [62] | 1999 | Shengxu Wang | Journal of Guangzhou University of Traditional Chinese Medicine | Influence of Electroacupuncture on Spinal Monoamine Neurotransmitter Content in Rats with Adjuvant Arthritis (in Chinese) | basic research | Serotonin  Dopamine Norepinephrine |
| [63] | 1995 | Yong Huang | Chinese Journal of Traditional Medical Science and Technology | An experimental study on the effect of electroacupuncture at *'Zusanli'* at different times on pain threshold, monoamine mediators, and histamine in adjuvant arthritis rabbits (in Chinese) | basic research | Dopamine  Histamine |
| [64] | 1998 | Yong Huang | Journal of Chengdu University of Traditional Chinese Medicine | Observation of diurnal rhythm of plasma 5-HIAA in adjuvant arthritis rabbits and the effect of acupuncture on it (in Chinese) | basic research | 5-HIAA |
| [65] | 2003 | Rong Luo | Chengdu University of Traditional Chinese Medicine | Research on the Mechanism of Action of the Sympathetic Nervous System in the Treatment of Experimental RA with Moxibustion (in Chinese) | basic research | Epinephrine |
| [66] | 2018 | Jianxin Xu | Chinese Journal of Traditional Medical Science and Technology | Clinical observation of electroacupuncture treatment for rheumatoid arthritis (in Chinese) | Clinical research | Norepinephrine Epinephrine Serotonin |
| [67] | 1985 | [Caiyuan](https://kns.cnki.net/kcms2/author/detail?v=hx6LgM6qJjvrOQNJGFhvFj-e3fsnw-_OkQ-qeFKrrVa2E0b9IGWJ0_syFPw-n9loCltdS5uq2JQlhtq0OjccrkzUYg5WpBIOw-pv8GBpfesoILeMLjpk1WMinfLYPxmR&uniplatform=NZKPT&language=CHS) Wang | Acupuncture Research | The interrelationship between methionine-enkephalin (Met-Enk) and acetylcholine (ACh) in the brain of rats and their effects on electroacupuncture analgesia. (in Chinese) | basic research | MET-enkephalin  ACh |

Abbreviations: CCK-8, Cholecystokinin-8; 5-HIAA,5-Hydroxyindole-3-acetic acid; ACH, acetyl choline; CGRP, Calcitonin Gene-Related Peptide; SP, substance P; VIP, Vasoactive Intestinal Peptide; ACTH, Adrenocorticotropic hormone; CRH, Corticotropin-releasing hormone; HVA, High vanillic acid; DOPAC,3,4-Dihydroxyphenylacetic acid;

**REFERENCES**

1. Shou Y, Yang Y, Xu M-S, Zhao Y-Q, Ge L-B, Zhang B-M. Electroacupuncture inhibition of hyperalgesia in rats with adjuvant arthritis: involvement of cannabinoid receptor 1 and dopamine receptor subtypes in striatum. Evidence‐Based Complementary Alternative Medicine.2013;2013(1):393460.

2. Yoo Y-C, Oh JH, Kwon TD, Lee YK, Bai SJJYmj. Analgesic mechanism of electroacupuncture in an arthritic pain model of rats: a neurotransmitter study. 2011;52(6):1016.

3. Huang M, Zhang D, Sa Z-y, Xie Y-y, Gu C-l, Ding G-h. In Adjuvant‐Induced Arthritic Rats, Acupuncture Analgesic Effects Are Histamine Dependent: Potential Reasons for Acupoint Preference in Clinical Practice. Evidence‐Based Complementary Alternative Medicine.2012;2012(1):810512.

4. Seo B-K, Sung W-S, Park Y-C, Baek Y-H. The electroacupuncture-induced analgesic effect mediated by 5-HT1, 5-HT3 receptor and muscarinic cholinergic receptors in rat model of collagenase-induced osteoarthritis. BMC complementary alternative medicine.2016;16(1):212.

5. Yuan X-C, et al. Electroacupuncture potentiates cannabinoid receptor-mediated descending inhibitory control in a mouse model of knee osteoarthritis. 2018;11(112.

6. Zhu H, et al. Inhibition of GABAergic neurons and excitation of glutamatergic neurons in the ventrolateral periaqueductal gray participate in electroacupuncture analgesia mediated by cannabinoid receptor. Front. Neurosci. 13, 484. 2019.

7. Chai W, et al. Electroacupuncture alleviates pain responses and inflammation in a rat model of acute gout arthritis. Evidence‐based Complementary

Alternative Medicine.2018;2018(1):2598975.

8. Li A, et al. Corticosterone mediates electroacupuncture-produced anti-edema in a rat model of inflammation. BMC Complementary Alternative Medicine.2007;7(1):27.

9. Li A, et al. Electroacupuncture activates corticotrophin-releasing hormone-containing neurons in the paraventricular nucleus of the hypothalammus to alleviate edema in a rat model of inflammation. BMC Complementary Alternative Medicine.2008;8(1):20.

10. Yoichi M, Kazunori I, Kenji I, Hiroshi K. Effect of electroacupuncture on carrageenan-induced muscle pain in rats. Pain Research.2010;25(4):211-21.

11. Zhang Y-Q, Ji G-C, Wu G-C, Zhao Z-Q. Excitatory amino acid receptor antagonists and electroacupuncture synergetically inhibit carrageenan-induced behavioral hyperalgesia and spinal fos expression in rats. Pain.2002;99(3):525-35.

12. Zhang L-X, Li X-L, Wang L, Han J-S. Rats with decreased brain cholecystokinin levels show increased responsiveness to peripheral electrical stimulation-induced analgesia. Brain Res.1997;745(1-2):158-64.

13. Lee G-S, et al. Enhancement of electroacupuncture-induced analgesic effect in cholecystokinin-A receptor deficient rats. Brain Res Bull.2003;62(2):161-4.

14. Tang N-M, Dong H-W, Wang X-M, Tsui Z-C, Han J-S. Cholecystokinin antisense RNA increases the analgesic effect induced by electroacupuncture or low dose morphine: conversion of low responder rats into high responders. Pain.1997;71(1):71-80.

15. Huang C, Hu Z, Jiang S, Li H, Han J, Wan Y. CCKB receptor antagonist L365, 260 potentiates the efficacy to and reverses chronic tolerance to electroacupuncture-induced analgesia in mice. Brain Res Bull.2007;71(5):447-51.

16. Kim W, Kim SK, Min BI. Mechanisms of electroacupuncture-induced analgesia on neuropathic pain in animal model. Evid Based Complement Alternat Med.2013;2013(436913.

17. Tsai HY, Lin JG, Inoki R. Further evidence for possible analgesic mechanism of electroacupuncture: effects on neuropeptides and serotonergic neurons in rat spinal cord. Jpn J Pharmacol.1989;49(2):181-5.

18. Wang S, Hong J, Lai X. Influence of Electroacupuncture on Spinal Monoamine Neurotransmitter Content in Rats with Adjuvant Arthritis Journal of Guangzhou University of Traditional Chinese Medicine.2001;01):1-2+1

19. Xu J, Feng Q, Zhao C, Song K. The effect of electroacupuncture on C-fos expression in the hippocampus of rats with experimental rheumatoid arthritis. Chinese Acupuncture & Moxibustion.1998;01):47-9+4 (in chinese).

20. Wang S, Lai X, He H, Yang Z. Effect of Bilateral Paravertebral Electroacupuncture on Spinal Prodynorphin mRNA Expression in Rats with Adjuvant-Induced Arthritis Chinese Journal of Traditional Medical Science and Technology.1999;01):1-3+5 (in Chinese).

21. Wang S, Hong J, Lai X, Cai J. Immunohistochemical Evaluation of Electroacupuncture at Jiaji Points on Substance P Expression in the Spinal Cord of Rats with Freund’s Adjuvant-Induced Arthritis. Chinese Journal of Traditional Medical Science and Technology.2000;03):131-2+27 (in Chinese).

22. Wang，SX, Hong J, Zhou，YL, Lai X. Effect of Electroacupuncture at Jiaji Points on Peripheral Blood Monoamine Levels in Rats with Adjuvant-Induced Arthritis. Chinese Journal of Traditional Medical Science and Technology.2000;05):273-4+69 (in Chinese).

23. Yang J, Song K, Liang F, Zhao C, Yang J. The effect of electroacupuncture on the content of inflammatory mediators in the inflamed area of adjuvant arthritis rats. . Journal of Chengdu University of Traditional Chinese Medicine.1999;01):48-9

24. Huang D, Yu S, Han Z, Luo R, Hao L. The effect of wheat-grain moxibustion on local tissue inflammatory mediators in experimental RA rats. Shanghai Journal of Acupuncture and Moxibustion.2002;04):43-4 (in chinese).

25. Zhao C, Yang J, Song K, Luo Y, Liang F. The effect of electroacupuncture on local β-EP and LEK content in adjuvant arthritis rats. Journal of Basic Chinese Medicine.1999;08):59-61 (in chinese).

26. Gao X, Wang X, liu X, Jia M, Li H. The effects of different moxibustion methods on the hypothalamic-pituitary-adrenal axis function in rabbits with rheumatoid arthritis Journal of Basic Chinese Medicine.2015;21(09):1140-2.

27. Ma W, Liu X, Zhou H. Effects of Chronological Moxibustion on Circadian Rhythm Activities of Hypothalamus-Pituitary-Axis in Rheumatoid Arthritis Rats Acupuncture Research.2016;41(02):100-7 (in Chinese).

28. Yang J, Song K, Liang F, Zhao C, Yang J, Wang R. A study on the peripheral analgesic mechanisms of different acupuncture therapies in adjuvant-induced arthritis rats Chinese Acupuncture & Moxibustion.1999;06):43-7+5 (in Chinese).

29. Liu X, Huang D, Hao L, Song K, Yu S, Chen X. The effect of moxibustion on the hypothalamic-pituitary-adrenal axis in rats with rheumatoid arthritis Journal of Basic Chinese Medicine.2003;02):57-9 (in Chinese).

30. Wei J, Liu M, Lu S, Qiao X. The Difference of Effect on Plasma Corticosterone Content of Adjuvant-induced Arthritis Rats between Acupuncture and Moxibustion Therapy. Journal of Chengdu Medical College.2008;3(04):283-4 (in Chinese).

31. Liu X, et al. The impact of warming on the immune function of patients with rheumatoid arthritis. Shanghai Journal of Acupuncture and Moxibustion.1997;05):6-7 (in Chinese).

32. Jiang Y, He X, Yin X, Shen Y, Fang J. Anti-inflammatory and synovial-opioid system effects of electroacupuncture intervention on chronic pain in arthritic rats. Chinese Acupuncture & Moxibustion.2015;35(09):917-21 (in chinese).

33. Mu J, Liu H, Hu W, Jiang Z, Wang T. A study on the analgesic effect of acupuncture on experimental arthritis in rats Journal of Nanjing Medical University(Natural.1987;02):104-7+62-63 (in chinese).

34. S K, Sun S. The relationship between acupuncture for alleviating chronic pain syndrome and increased plasma methionine-enkephalin (MEK) concentration. Information on Traditional Chinese Medicine.1984;01):35-6 (in chinese).

35. Wang H, Fang J, Du J, Fu t, Wang L, Liang Y. A study on the correlation between hypothalamic enkephalin and its degradation enzyme content and the efficacy of electroacupuncture analgesia. . Zhejiang Journal of Traditional Chinese Medicine.2016;51(11):799-800 (in chinese).

36. Peng C, Hu L, Wu Z, He L, Fan R. The effect of acupuncture combined with moxibustion on TRPV1 expression in dorsal root ganglia of rats with rheumatoid arthritis. China Journal of Traditional Chinese Medicine and Pharmacyv.2021;36(12):7310-3 (in chinese).

37. Luo L. Research on the local mechanism of immune regulation by moxibustion [Doctor]: Chengdu University of Traditional Chinese Medicine; 2010.

38. Tang Z, et al. Experimental study on effects of moxibustion's anti inflammatory and immunity action on neurotransmitter Journal of Basic Chinese Medicine.2000;09):53-5 (in Chinese).

39. Junrong B. Clinical Effect of Huangqi Jianzhong Decoction Combined with Warm Acupuncture on Rheumatoid Arthritis (in Chinese) Chinese Medicine Modern Distance Education of China.2021;19(03):99-101.

40. Wanh F, Wan J, Zhang C, Pu Y, Ding X, Li J. Observation on the Efficacy of Warm Needling Moxibustion as Mainly in Treating Active Rheumatoid Arthritis Shanghai Journal of Acupuncture and Moxibustion.2019;38(07):779-83 (in Chinese).

41. Feng Y, Chang Z, Li Y. The effect of combined warm needle acupuncture and modified Taohong Yin on rheumatoid arthritis with blood stasis obstruction, as well as its impact on bone metabolism, MCP-1, and TGF-β1. Clinical Research and Practice.2023;8(32):106-9 (in chinese)

42. Li J, Sui M, Lin S, Pei w, Lin G. Evaluation of the effects of fireneedle on the levels of 5-HT, HA and PGE2 in rheumatoid arthritis rats by microdialysis. Journal of Jinan University (Natural Science & Medicine Edition).2017;38(04):314-21 (in Chinese).

43. Wang Y, Wu J, Jin H. Effects of eletroacupuncture on met-enkephalin, dynorphin in patient plasma and mouse spinal cord and analgesia Journal of Zhengzhou University (Medical Sciences).2001;04):425-6 (in Chinese).

44. Ma Z, et al. A clinical study on the impact of acupuncture on the immune-neuroendocrine system of patients with rheumatoid arthritis. Chinese Acupuncture & Moxibustion.1997;09):529-31 (in Chinese).

45. Luo F, et al. Repeated Electroacupuncture Modulates Spontaneous Release of Substance P, Cholecystokinin-Octapeptide, and Methionine-Enkephalin in Rats with Adjuvant-Induced Monoarthritis. Chinese Journal of Pain Medicine.1996;03):169-75 (in Chinese).

46. Wang C, Li L, Liu X, Guan X. The effects of central and peripheral acetylcholine on methionine-enkephalin in the rat brain and their interrelationship with electroacupuncture analgesia. Acta Medicinae Universitatis Scientiae et Technologiae Huazhong.1994;03):191-4 (in chinese).

47. Ji B, et al. Effects of acupotome dissolution therapy and electro-acupuncture on central monoamine neurotransmitters in rabbits with knee osteoarthritis Journal of Beijing University of Traditional Chinese Medicine.2009;32(09):598-602 (in Chinese).

48. Qiu N, Mu J, Zhang J, Liu H, Chen C. Influence of Analgesics and Acupuncture on Plasma Cyclic Adenosine Monophosphate Content in a Rat Model of Adjuvant-Induced Arthritis. Acupuncture Research.1988;04):350-3 (in Chinese).

49. Liu X, Liu C, Li Q, Sun L, Xiao J, Yin S. The impact of warming acupuncture on beta-endorphin, interleukin-2, and NK cell activity in the serum of rheumatoid arthritis patients. Academic Journal of Chinese PLA Medical School.1997;02):11-2 (in Chinese).

50. Ji B, Guo C, Jin Y, Zhong D, Chen Y, Guo M. Effects of acupotome and electro-acupuncture on pain threshold and monoamine-neurotransmitters of central nerves in rats with knee osteoarthritis. Chinese Journal of Pathophysiology.2010;26(06):1091-5 (in Chinese).

51. Li X, et al. Study on Effect and Mechanism of Analgesia on Adjuvant-induced Arthritis Rats with Crossing Selection of Acupoints Method. Journal of Practical Traditional Chinese Medicine.2007;10):615-6 (in Chinese).

52. Tan Q, Li J, Li J, Li B, Xiang H, Cai G. Effect of Acupuncture at Zusanli(ST 36) on Gastrointestinal Motility in Rats with Collagen-induced Arthritis. World Chinese Medicine.2022;17(09):1278-82 (in Chinese).

53. Yiin S, Ma Z, Liu X, Sun L, Xiao J. Effects of Warm Needling on the Concentrations of PlasmaPostaglandin E, in the Patients with Rheumatoid Arthritis. Acupuncture Research.1997;04):280-2 (in Chinese).

54. Han J, Wang J, Yu X, Wu F. Effect of Electroacupuncture on the Expression of Adenosine A1 Receptor in Hypothalamus and Spinal Cord of Adjuvant Arthritis Rats. Shanghai Journal of Acupuncture and Moxibustion 2016;35(01):71-5 (in Chinese).

55. Hao L. The regulatory mechanism of moxibustion on the neuroimmuneendocrine network in experimental RA rats [Doctor]: Chengdu University of Traditional Chinese Medicine; 2001.

56. Xiaorong C, Kun A, Li L, Meirong L. The Im pact of Electroacupunc turng on PontZusanlj Guanyuan toβ -EP, ACTH ofAdjuvantnduced A rhritis Rats Chinese Archives of Traditional Chinese Medicine.2009;27(04):685-7 (in Chinese).

57. Li H, Li X, Zhang L, Zhu W, Zhai J, Zhou D. The effect of electroacupuncture on the content of CRH, IL-2, and β-EP in the hypothalamus of adjuvant arthritis rats. Chinese Acupuncture & Moxibustion.2005;11):45-8 (in chinese).

58. Ren C, Du X, Fang X. Analgesic Effect of Buccal Acupuncture and Changes of β-EP and CCK-8 in Cerebrospinal Fluid of Experimental Rheumatoid Arthritis Rabbits Chinese Journal of Information on Traditional Chinese Medicine.2012;19(12):37-9 (in Chinese).

59. Jie W, et al. Analgesic effect of buccal acupuncture on acute arthritis in rabbits and underlying mechanisms. Journal of Central South University (Medical Science).2017;42(05):517-21 (in Chinese).

60. Xu M, Yang C, Shi T, Yang W. A study on the mechanism of how levels of cholecystokinin octapeptide within the brain influence the efficacy of acupuncture analgesia. Chinese Journal of Tissue Engineering Research.2003;31):4233-5 (in chinese).

61. Huang Y, Yang J, Song K. Observation of circadian rhythms of plasma dopamine in adjuvant arthritis rabbits and the effect of acupuncture on it. Chinese Journal of Traditional Medical Science and Technology.1998;03):131-2 (in Chinese).

62. Wang S, Hong J, Zhou Y, Lai X. The Influence of Electro-Acupuncture on Jiaji Points on The Content of Spinal Cords' Monoamine Neurotransmittttter Arihritis Rats Induced By Adjuvant. Journal of Guangzhou University of Traditional.1999;04):286-8 (in chinese).

63. Huang Y. An experimental study on the effect of electroacupuncture at 'Zusanli' at different times on pain threshold, monoamine mediators, and histamine in adjuvant arthritis rabbits. Chinese Journal of Traditional Medical Science and Technology.1995;01):9-10 (in Chinese).

64. Huang Y, Yang J, Song K. Observation of diurnal rhythm of plasma 5-HIAA in adjuvant arthritis rabbits and the effect of acupuncture on it. Journal of Chengdu University of Traditional Chinese Medicine.1998;01):31-2+57 (in Chinese).

65. Luo R. Research on the Mechanism of Action of the Sympathetic Nervous System in the Treatment of Experimental RA with Moxibustion [Doctor]: Chengdu University of Traditional Chinese Medicine; 2003.

66. Xu J. Clinical observation of electroacupuncture treatment for rheumatoid arthritis. Chinese Journal of Traditional Medical Science and Technology.2018;25(06):914-6 (in Chinese).

67. Wang C, et al. The interrelationship between methionine-enkephalin (Met-Enk) and acetylcholine (ACh) in the brain of rats and their effects on electroacupuncture analgesia. Acupuncture Research.1985;02):119-24+91 (in chinese).
