# Supplementary material for: Potential mechanisms of acupuncture treatment for rheumatoid arthritis: a study based on network topology and machine learning
Source: Chin Med. 2025 Oct 7;20:164. doi: 10.1186/s13020-025-01209-8 (PMC12502209; doi:10.1186/s13020-025-01209-8)
Supplement: Supplementary file 4 — Additional file 4. [file 13020_2025_1209_MOESM4_ESM.docx]

| **Type** | **Bioactive components** | **Citation Frequency** |
| --- | --- | --- |
| Neurotransmitters/Neuromodulators | Histamine | 7 |
|  | Dopamine | 10 |
|  | Norepinephrine | 11 |
|  | Epinephrine | 6 |
|  | Serotonin | 16 |
|  | acetyl choline (ACH) | 2 |
|  | Glutamate | 1 |
|  | GABA | 1 |
|  | Adenosine | 1 |
|  |  |  |
| Neurotransmitter metabolites | 5-Hydroxyindole-3-acetic acid(5-HIAA) | 7 |
|  | 3,4-Dihydroxyphenylacetic acid (DOPAC) | 1 |
|  | High vanillic acid (HVA) | 1 |
|  |  |  |
| Neuropeptide | β--endorphin | 9 |
|  | Deltorphin | 3 |
|  | MET-enkephalin | 10 |
|  | L-enkephalin | 7 |
|  | Cholecystokinin-8 (CCK-8) | 9 |
|  | Vasoactive Intestinal Peptide (VIP) | 1 |
|  | Calcitonin Gene-Related Peptide (CGRP) | 1 |
|  | substance P(SP) | 4 |
|  |  |  |
| Inflammatory Mediator | Dinoprostone | 6 |
|  |  |  |
| Hormones (regulators of the neuroendocrine system) | Adrenocorticotropic hormone (ACTH) | 6 |
|  | Corticotropin-releasing hormone (CRH) | 3 |
|  | Corticosterone | 8 |
