# Supplementary material for: Potential mechanisms of acupuncture treatment for rheumatoid arthritis: a study based on network topology and machine learning
Source: Chin Med. 2025 Oct 7;20:164. doi: 10.1186/s13020-025-01209-8 (PMC12502209; doi:10.1186/s13020-025-01209-8)
Supplement: Supplementary file 5 — Additional file 5. [file 13020_2025_1209_MOESM5_ESM.docx]

**Supplementary Table 3** Core gene information table

| Gene | Target name | Degree | Betweenness  Centrality | Closeness  Centrality |  |
| --- | --- | --- | --- | --- | --- |
| TNF | TNF-alpha | 161 | 0.096666464 | 0.803030303 |  |
| IL6 | Interleukin-6 | 159 | 0.085050973 | 0.796992481 |  |
| GAPDH | Glyceraldehyde-3-phosphate dehydrogenase liver | 145 | 0.092121031 | 0.759856631 |  |
| AKT1 | RAC-alpha  serine/threonine-protein kinase | 132 | 0.037130241 | 0.726027397 |  |
| ALB | Albumin | 128 | 0.038155114 | 0.716216216 |  |
| EGFR | Epidermal growth factor receptor erbB1 | 119 | 0.026959905 | 0.692810458 |  |
| SRC | Proto-oncogene  tyrosine-protein kinase Src | 112 | 0.059265308 | 0.670886076 |  |
| MMP9 | Matrix metalloproteinase 9 | 111 | 0.017198675 | 0.673015873 |  |
| CASP3 | Caspase-3 | 107 | 0.013883887 | 0.668769716 |  |
| MAPK3 | MAP kinase ERK1 | 103 | 0.018264171 | 0.658385093 |  |
| BCL2 | Apoptosis regulator Bcl-2 | 101 | 0.012422517 | 0.656346749 | |
| HSP90AA1 | Heat shock protein HSP 90-alpha | 99 | 0.019130976 | 0.650306748 | |
| PTGS2 | Cyclooxygenase-2 | 97 | 0.017415086 | 0.6443769 | |
| IGF1 | Insulin-like growth factor I | 92 | 0.008888106 | 0.632835821 | |
| IL2 | Interleukin-2 | 91 | 0.021786218 | 0.634730539 | |
| ESR1 | Estrogen receptor alpha Peroxisome | 91 | 0.009970287 | 0.630952381 | |
| PPARG | proliferator-activated receptor gamma | 88 | 0.012948369 | 0.629080119 | |

continued table

| Xubiao xubiao  HSP90AB1 | Heat shock protein HSP  90-beta | 88 | 0.014277799 | 0.629080119 |
| --- | --- | --- | --- | --- |
| MMP2 | Matrix metalloproteinase-2 | 85 | 0.010050396 | 0.619883041 |
| KDR | Vascular endothelial growth factor receptor 2 | 77 | 0.005629835 | 0.602272727 |
| MTOR | Serine/threonine-protein kinase mTOR | 75 | 0.003787829 | 0.603988604 |
| STAT1 | Signal transducer and activator of transcription 1-alpha/beta | 74 | 0.012033414 | 0.598870056 |
| ANXA5 | Annexin A5 | 71 | 0.004711979 | 0.598870056 |
| MAPK1 | MAP kinase ERK2 | 68 | 0.007260776 | 0.588888889 |
| CRP | C-reactive protein | 68 | 0.009876184 | 0.588888889 |
| RHOA | Transforming protein RhoA | 68 | 0.007103738 | 0.587257618 |
| PIK3CA | PI3-kinase p110-alpha subunit | 68 | 0.002986632 | 0.584022039 |
| KIT | Stem cell growth factor receptor | 66 | 0.005181828 | 0.584022039 |
| HRAS | GTPase Hras | 65 | 0.003254083 | 0.580821918 |
| JAK2 | Tyrosine-protein kinase JAK2 | 64 | 0.002450943 | 0.582417582 |
| PIK3R1 | Phosphatidylinositol 3-kinase regulatory subunit alpha | 64 | 0.004594061 | 0.577656676 |
| ITGB1 | Integrin alpha-4/beta-1 | 63 | 0.004910132 | 0.579234973 |
| BCL2L1 | Bcl-2-like protein 1 | 63 | 0.002535422 | 0.584022039 |

continue table

| CCL5 | C-C motif chemokine 5 | 63 | 0.004918622 | 0.582417582 |
| --- | --- | --- | --- | --- |
| MDM2 | E3 ubiquitin-protein ligase Mdm2 | 61 | 0.002561563 | 0.579234973 |
| HGF | Hepatocyte growth factor | 61 | 0.002611393 | 0.574525745 |
| MAPK8 | Mitogen-activated protein kinase 8 | 61 | 0.004045637 | 0.579234973 |
| SIRT1 | NAD-dependent deacetylase sirtuin 1 | 60 | 0.010508484 | 0.577656676 |
| IGF1R | Insulin-like growth factor I receptor | 60 | 0.00165359 | 0.577656676 |
| MPO | Myeloperoxidase | 58 | 0.006768008 | 0.569892473 |
| MAPK14 | MAP kinase p38 alpha | 56 | 0.003178819 | 0.568364611 |
| MET | Hepatocyte growth factor receptor | 56 | 0.001971332 | 0.565333333 |
| PARP1 | Poly [ADP-ribose] polymerase-1 | 56 | 0.003271933 | 0.572972973 |
| MET | Hepatocyte growth factor receptor | 56 | 0.001971332 | 0.565333333 |
| PARP1 | Poly [ADP-ribose] polymerase-1 | 56 | 0.003271933 | 0.572972973 |
| SYK | Tyrosine-protein kinase SYK | 56 | 0.005458674 | 0.56684492 |
| CASP1 | Caspase-1 | 56 | 0.003153977 | 0.571428571 |
| HMOX1 | Heme oxygenase 1 | 54 | 0.001797631 | 0.56684492 |
| PLG | Plasminogen | 53 | 0.005610676 | 0.563829787 |
| PTPN11 | Protein-tyrosine phosphatase 2C | 53 | 0.002823111 | 0.560846561 |
| CASP8 | Caspase-8 | 53 | 8.35E-04 | 0.568364611 |

continued table

| LCK | Tyrosine-protein kinase LCK | 53 | 0.005292585 | 0.565333333 |
| --- | --- | --- | --- | --- |
| ACE | Angiotensin-converting enzyme | 52 | 0.003442815 | 0.560846561 |
| PGR | Progesterone receptor | 52 | 0.002078793 | 0.560846561 |
| CTSB | Cathepsin B | 50 | 0.011572135 | 0.554973822 |
| NOS3 | Nitric oxide synthase 3 | 50 | 0.002506047 | 0.562334218 |
| MMP3 | Matrix metalloproteinase 3 | 50 | 0.002974654 | 0.554973822 |
| CDK2 | Cyclin-dependent kinase 2/cyclin A | 50 | 0.00307543 | 0.557894737 |
| MMP1 | Matrix metalloproteinase 1 | 49 | 0.002283444 | 0.559366755 |
| SELE | Selectin E | 49 | 0.00223119 | 0.556430446 |
| NR3C1 | Glucocorticoid receptor | 48 | 0.003420585 | 0.559366755 |
| FLT1 | Vascular endothelial growth factor receptor 1 | 48 | 0.001205488 | 0.554973822 |
| AR | Androgen Receptor | 48 | 0.002515989 | 0.557894737 |
| JAK1 | Tyrosine-protein kinase JAK1 | 48 | 0.001100706 | 0.552083333 |
| REN | Renin | 47 | 0.002516298 | 0.556430446 |
| LGALS3 | Galectin-3 | 46 | 0.001690845 | 0.552083333 |
| PLAU | Urokinase-type plasminogen activator | 45 | 0.001702121 | 0.552083333 |
